# The role of TGR5 as an onco-immunological biomarker in tumor staging and prognosis by encompassing the tumor microenvironment

**DOI:** 10.3389/fonc.2022.953091

**Published:** 2022-10-20

**Authors:** Zhiyuan Guan, Liying Luo, Shengfu Liu, Zhiqiang Guan, Qinggang Zhang, Zhong Wu, Kun Tao

**Affiliations:** ^1^ Department of Orthopedics, The Shanghai Tenth People’s Hospital of Tongji University, Shanghai, China; ^2^ Department of Ophthalmology, Tongren Hospital, Shanghai Jiao Tong University School of Medicine, Shanghai, China; ^3^ Department of Dermatology, Xuzhou Municipal Hospital Affiliated With Xuzhou Medical University, Xuzhou, China

**Keywords:** TGR5, cancer, prognosis, immune, methylation, survival

## Abstract

The relationship between G protein–coupled bile acid receptor 1 (TGR5, GPBAR1) and, specifically, cancer has been studied in *in vivo* and *in vitro* experiments, but there is still a lack of pan-cancer analysis to understand the prognostic significance and functioning mechanism of TGR5 in different cancer-driving oncogenic processes. Here, we used Gene Expression Integration, Human Protein Atlas, and The Cancer Genome Atlas (TCGA) to perform a pan-cancer analysis of the role of TGR5 in all 33 tumors. In all TCGA tumors, the TGR5 gene expression has been assessed, and we found that the high TGR5 gene expression in most cancers is associated with poor prognosis of overall survival for cancers such as glioblastoma multiforme (p = 0.0048), kidney renal papillary cell carcinoma (p = 0.033), lower grade glioma (p = 0.0028), thymoma (p = 0.048), and uveal melanoma (p = 0.004), and then the lower expression of TGR5 was linked with poor prognosis in cervical squamous cell carcinoma and endocervical adenocarcinoma (p = 0.014), malignant mesothelioma (MESO) (p = 0.048), sarcoma (p = 0.018), and skin cutaneous melanoma (p = 0.0085). The TGR5 expression was linked with the immune infiltration level of the macrophage M2_TIDE and was also associated with DNA methylation in ovarian and breast cancers. The regulation of hormone secretion, Rap1 pathway, osteoclast differentiation, and bile acid pathway was involved in the functional mechanism of TGR5. Besides, gene expressions were different in different tumors detected by RT-PCR, and cell activity experiments have also found that TGR5 can increase the activity of renal cell carcinoma and reduce the activity of skin cancer and osteosarcoma cells. In this investigation, the aim was to assess the comprehensive overview of the oncogenic roles of TGR5 in all TCGA tumors using pan-analysis.

## Highlights

- A first pan-cancer analysis of TGR5
- The TGR5 gene was highly expressed in most tumors that were associated with different prognoses.
- The macrophage M2_TIDE and DNA methylation may associate with the expression of TGR5.
- The correlation between the oncogenic role of TGR5 and the regulation of hormone secretion, Rap1 signaling pathway, and osteoclast differentiation may play an essential role in the progression of cancer.

## Introduction

In recent years, the therapeutic advances of cancer immunotherapy have developed rapidly and achieved remarkable effectiveness in treating a broad range of cancers by the interaction between the immune system and human cancers. Despite the successful application of cancer immunotherapy across various types of human cancers, the majority of patients with tumor have limited or no response to these treatments. Therefore, there is a desperate need in excavating predictive biomarkers to assess the response to those immunotherapy approaches and defining staging and prognosis at an early stage. Previous studies have discovered a few indicators associated with tumor prognosis and progression; however, accurate biomarkers for predicting clinical outcome and patient survival continue to be explored.

TGR5 (G protein–coupled bile acid receptor1), also known as GPBAR1, belonging to the G protein–coupled receptor (GPCR) superfamily (1), is activated by primary and secondary bile acids and expressed in different liver cells. GPBAR1 is a critical component of extracellular which can activates downstream AKT, NF-kB, and extracellular signal–regulated kinases 1/2 pathways and exchanges protein directly by an activated cAMP (Epac) pathway (2–7). Our previous research found that the activation of TGR5 mediates dermatitis and osteoporosis by a downregulated JAK1-STAT3 signaling pathway (8). Lines of evidence have been accrued on the role that TGR5 is related to different cancers such as gastric cancer (9), endometrial cancer (10), esophageal adenocarcinoma (11), cell lung cancer (12), hepatic carcinoma (13, 14), and colorectal cancer (15). Previous studies, however, have limited the role of TGR5 in a specific type of tumor. Considering the complicated and distinctive relationship between TGR5 and tumors, it is elusive to investigate the potential of TGR5 to serve as a predictive biomarker in all The Cancer Genome Atlas (TCGA) tumors.

Because molecular aberration and the complexity of tumorigenesis have increased unprecedentedly in scale and accessibility, pan-cancer analysis has become very crucial in analyzing the clinical outcome and mechanism. The genomics information of different tumors contained in publicly funded TCGA projects and available Gene Expression Omnibus (GEO) databases allows us to analyze pan-cancer (16–18). Thus, to the best of our knowledge, this is the first research to use pan-cancer analysis of TGR5 across various tumor types and investigate the comprehensive overview of its potential role as an onco-immunological biomarker in tumor staging and prognosis.

In this study, we represented the TCGA and GEO databases to generate TGR5 detection to determine gene expression and DNA methylation, immune system infiltration, and cell signaling pathways across 33 various cancers. Furthermore, we also analyzed the molecular mechanism of TGR5 in the skin and renal cancer cell lines. Our study preliminarily unveiled the application of TGR5 as a predictive biomarker of tumor staging and prognosis, which warrants further investigation.

## Materials and methods

### Gene expression analysis

We used the Tumor Immune Estimate Resource (version 2; TIMER2) website (http://timer.cistrome.org/) and the ONCOMINE database (www.oncomine.org) to analyze the relationship between different tumor tissues and adjacent normal tissues, as well as the TGR5 expression in tumors (17). The threshold is defined as a p-value of 0.001 and a twofold change of 1.5. We used the Gene Expression Profiling Interactive Analysis (version 2; GEPIA2) (http://gepia2.cancer-pku.cn/#analysis) to acquire box plots of the Genotype-Tissue Expression (GTEx) database under the settings of p-value cutoff = 0.01, log2FC (fold change) cutoff = 1, and “match TCGA normal and GTEx data.” The total isoform usage changes of TGR5 in 33 types of tumors have also been analyzed in the GEPIA2 tool. The expression of TGR5 in the different pathological stages of all TCGA tumors had been investigated in the HEPIA2 tool. Mutation of the GPBAR1 gene was documented by oncogenomic analysis.

### Survival prognosis analysis

GEPIA is a web tool that provides key interactive analysis and customization capabilities, including tumor/normal differential expression profilometry, profile mapping, pathological staging, patient survival analysis, similar gene assay analysis, and dimensionality reduction analysis. We used GEPIA to address the differential expression of TGR5 in all common cancers. We applied the following cutoff criteria: using the ANOVA method, |log2FC| > 1, p-value < 0.01, and log2[TPM (transcripts per million) + 1] for log scale, matching TCGA and GTEx normal data, and adding all cancer tissue names.

We also used the GEPIA2 tool to analyze overall survival (OS) saliency maps and disease-free survival (DFS) of TGR5 survival data in all tumors. Cutoff values (50% and 50%) were used as expression thresholds to separate the high-expression and low-expression cohorts, and the logarithmic sequence test was used in the hypothesis test (19).

### Genetic alteration analysis

The cBioPortal tool (https://www.cbioportal.org/) was applied to analyze the protein structure, mutation type, mutation site information, copy number change (CAN), and the three-dimensional structure frequency of changes by checking all TCGAs.

### Immune infiltration analysis

The TIMER2 instrument was used to investigate the association between the TGR5 expression and the immune system infiltration in all TCGA tumors. Cancer-related fibroblasts, CD4 T cells, CD8 T cells, T-cell regulators, and macrophages were selected for detailed analysis.

### TGR5-related gene enrichment analysis

We used the protein–protein interaction network to study the interaction between protein names (TGR5) and organisms (*Homo sapiens*) in the STRING website (https://string-db.org/). The most important parameters are defined as follows: the minimum required interaction rating [low confidence (0.150)], the effectiveness of the network edge, and the maximum number of interactions to be displayed (no more than 50 first shells) and active interaction sources.

We obtained violin plots of the TGR5 expression in different pathological stages (stage I, stage II, stage III, and stage IV) of all TCGA tumors *via* the “Pathological Stage Plot” module of HEPIA2. The log2(TPM + 1) transformed expression data were applied for the box or violin plots.

The GEPIA2 tool was used to select the top 100 TGR5-related genes from all TCGA data sets based on tumors and normal tissues. Then, we performed a Pearson gene correlation analysis on TGR5 and selected genes, calculated the p-value and correlation coefficient (R), and displayed them in the corresponding fields of the graph. The heat map of the selected gene expression profile showed the partial correlation (color) and p-value in the Spearman degree correlation test after purity correction.

We used the Draw Venn Diagram, an interactive Venn diagram viewer, to contribute an intersection analysis between TGR5-binding and interacted genes. Then, the two sets of data were merged and filtered to perform a Kyoto Encyclopedia of Genes and Genomes (KEGG) pathway analysis. The enriched pathways were visualized with the “tidyr” and “ggplot2” R packages. The R language software (R-3.6.3, 64-bit) (https://www.r-project.org/) was used in this analysis. Two-tailed p < 0.05 was considered to be statistically significant (20).

### Immunohistochemical staining

In order to understand the expression level of the TGR5 protein, we performed immunohistochemical staining of the TGR5 protein in colorectal cancer, breast cancer, lung cancer, and pancreatic cancer and then analyzed these data using the Human Protein Atlas (HPA; https://www.proteinatlas.org/).

### DNA methylation analysis

We used MEXPRESS (https://mexpress.be/) and SMART network (http://www.bioinfo-zs.com/smartapp/) to analyze the DNA methylation levels of different TGR5 probes in all TCGA tumors. We also examined the methylation of the CpG aggregation of the TGR5 gene, the correlation of DNA methylation, the change of copy number variation, and different tumor stages. Take the beta value of each sample, and take the p correlation value of the Pearson correlation coefficient (R) according to Benjamin–Hochberg. MethSurv data (https://biit.cs.ut.ee/methsurv/) were also used to predict the methylation level of TGR5 DNA.

### Cell culture, transfection, and quantitative real-time PCR

Human skin basal cell carcinoma cell lines (BCC77015, USA) were grown in Dulbecco’s modified eagle medium (DMEM) (Thermo, USA) containing 10% FBS (Gibco, Los Angeles, CA) in an incubator with 5% CO_2_ at 37°C in six-well plates. Human osteosarcoma cell lines (MG-63, Shanghai, China) and human renal cancer cell lines (Caki-1, Shanghai, China) were cultured in McCoy’s 5a medium (Gibco, Los Angeles, CA) containing 10% FBS, ampicillin, and streptomycin at 37°C in a humidified atmosphere of 95% air and 5% CO_2_. Human renal cancer cell lines (786-O, Shanghai, China) were purchased from the Institute of Cell Biology, Chinese Academy of Sciences.

All cell lines have been used for more than 20 generations. The middle part was replaced the day before transfection. When the cells reach 70%–90% growth density, the cells were treated with TGR5 agonist (INT777) and si-TGR5 Sirna synthetic transfection (LipofectamineTM2000 Transfection Kit, Invitrogen, USA). At the same time, non-interfering siRNA transfection was used as a negative control. The transfection process was carried out in strict accordance with the kit instructions, and the cells transfected for 48 h were collected for the next step of detection.

As mentioned above, the Cell Counting Kit-8 (CCK-8) was used to assess cell viability (21). MG-63 BCC cells were seeded in 96-well plates. After cell adhesion, the cells were treated with 3-MA (5 mm) or without 3-MA for 2 h and treated next with cisplatin, doxorubicin, and methotrexate for the specified concentration and time. Then, we added 10 µl of CCK-8 reagent to each well, incubated it for 1 h at 37°C, and determined the relative number of viable cells by measuring the optical density of the cell lysate at 450 nm.

Total RNA was extracted from cells using TRIzol (Invitrogen). qRT-PCR was performed in triplicate using SYBR Green reagents (Lisheng, Inc., Shanghai, China) with specific primers for TGR5 (5′-cactgttgtccctcctctcc-3′ and reverse primer, 5′-acactgctttggctgcttg-3′) and glyceraldehyde-3-phosphate dehydrogenase (GAPDH forward primer, 5′-gaaggtgaaggtcggagt-3′ and reverse primer, 5′-catgggtggaatcatattggaa-3′).

### Statistical analysis

All measured values are expressed as mean ± standard deviation, and a p value of ≤ 0.05 is considered statistically significant. A repeated-measures two-way ANOVA was used in this study. GraphPad Prism 8.02 (La Jolla, California, USA) was used to analyze the data using one-way ANOVA and Tukey’s multiple.

## Results

### TGR5 differentially expressed between normal and tumor samples in 14 cancer types

**Figure 1:**
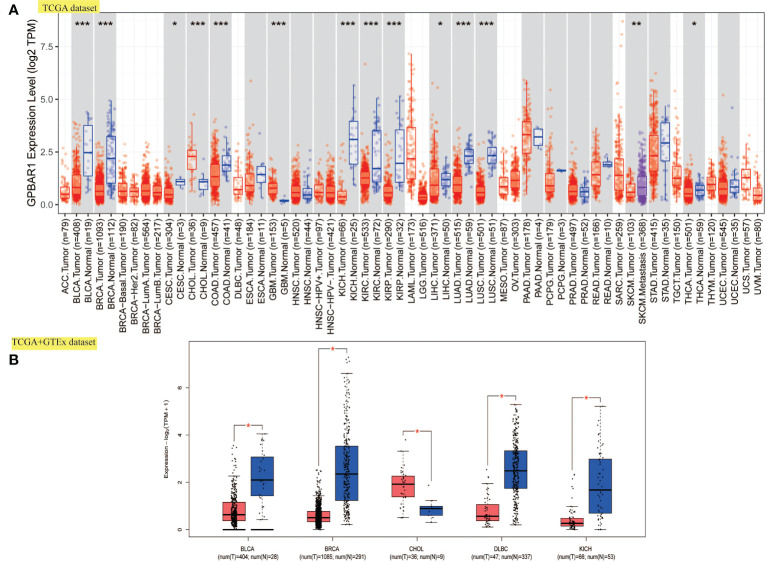
The expression level of TGR5 in all TCGA tumors. **(A)** The expression level of the TGR5 gene in various tumors and normal tissues. **(B)** Box plot expression of the TGR5 gene in BLCA, BRCA, CHOL, DLBC, and KICH from the TCGA and GTEx data sets. *p <.05, **p <.01, ***p <.001.

**Figure 2:**
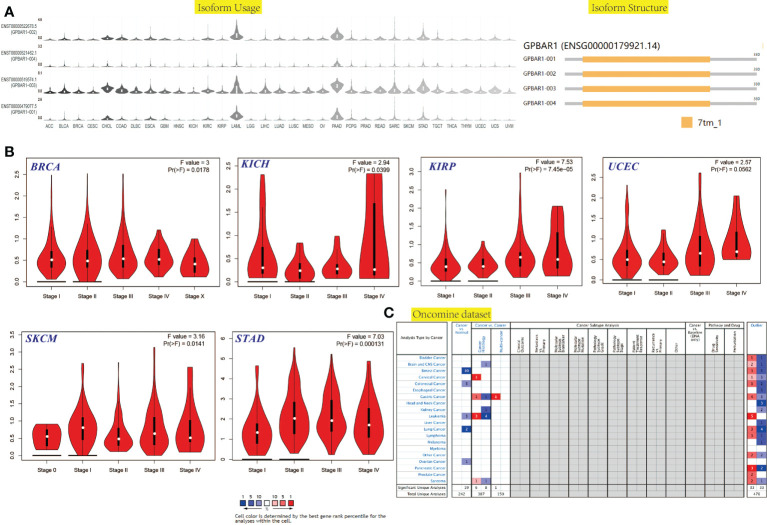
The isoform usage and structure, stage-dependent expression, mRNA level in the cancer-wide range of the TGR5 gene in all TCGA tumors. **(A)** The isoform usage and structure of the TGR5 gene in all TCGA tumors. **(B)** The stage-dependent expression of the TGR5 gene in BRCA, KICH, KIRP, UCEC, SKCM, and STAD were analyzed by the TCGA data set. **(C)** The mRNA level in the cancer-wide range of the TGR5 gene in all TCGA tumors from Oncomine data sets.

We first investigated the TGR5 expression across TCGA tumors based on the TIMER2 tool. **Figure 1A** showed a higher level of TGR5 gene expression in bladder urothelial carcinoma (BLCA), breast invasive carcinoma (BRCA), kidney chromophobe (KICH), colon adenocarcinoma (COAD), lung adenocarcinoma (LUAD), lung squamous cell carcinoma (LUSC), kidney renal clear cell carcinoma (KIRC), kidney renal papillary cell carcinoma (KIRP) (p < 0.001), skin cutaneous melanoma (SKCM) (p < 0.01), liver hepatocellular carcinoma (LIHC), cervical squamous cell carcinoma and endocervical adenocarcinoma (CESC), and thyroid carcinoma (THCA) (p < 0.05) and a lower level of TGR5 gene expression in cholangiocarcinoma (CHOL) and glioblastoma multiforme (GBM) (p < 0.001) than in the corresponding normal tissues. Alternative transcription subtypes usually serve as potential cancer stimulants in oncology **Figure 1B** (22). As shown in **Figure 2**, we analyzed the isoform usage and isoform structure of TGR5 and topics related to TGR5 expression and tumor staging including BRCA, KICH, KIRP, uterine corpus endometrial carcinoma (UCEC), SKCM, and gastric adenocarcinoma [stomach adenocarcinoma (STAD)] (**Figure 2B**, all ps < 0.05).

**Figure 3:**
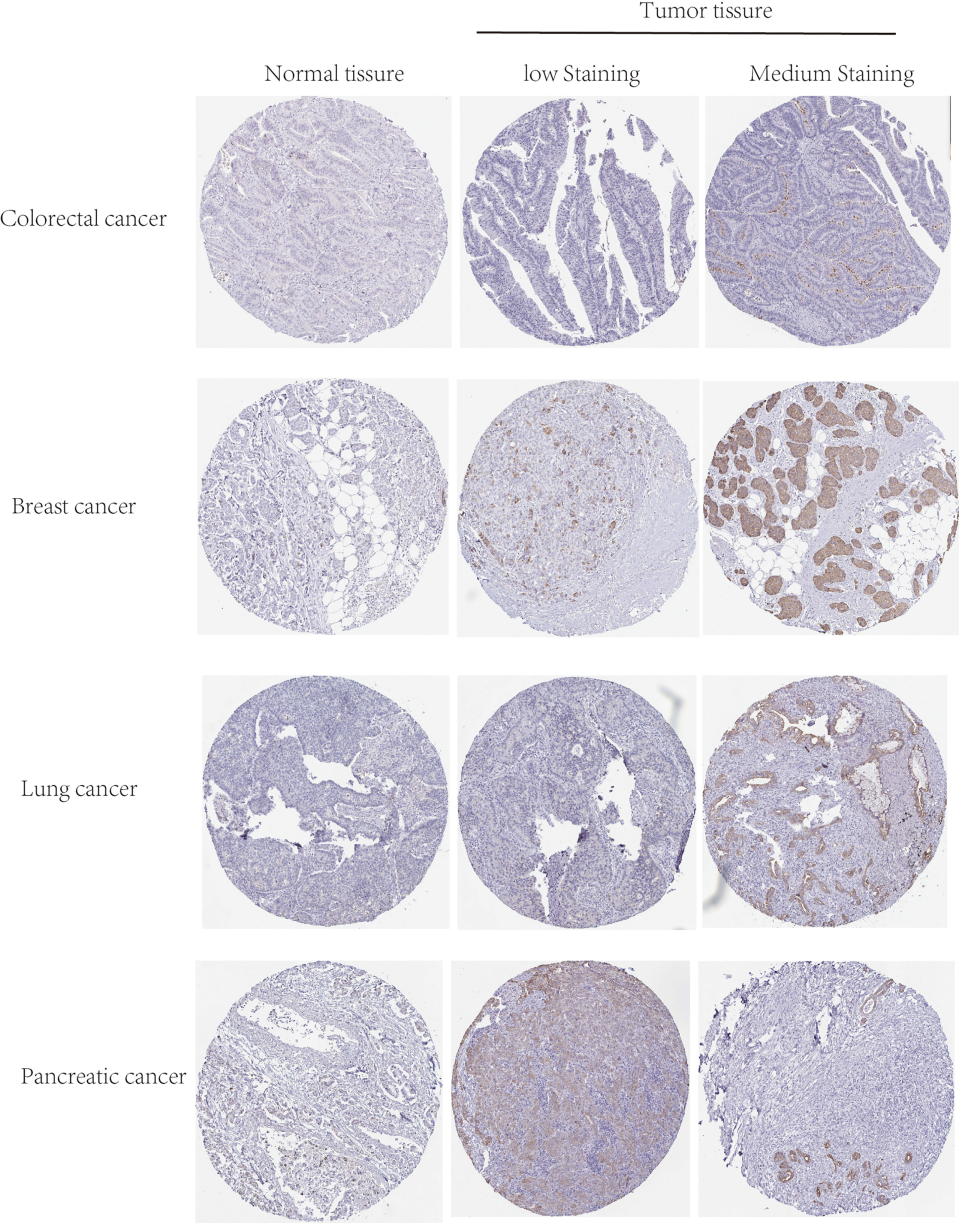
The protein level of TGR5 between normal, low, and medium staining in colorectal, breast, lung, and pancreatic cancers by immunohistochemistry images.

The gene expression of TGR5 was analyzed in the Oncomine data set over a cancer-wide range (**Figure 2C**). The pooling analysis also revealed that TGR5 is highly expressed in lymphoma, leukemia, colorectal cancer, gastric cancer, lung cancer, prostate cancer, brain cancer, breast cancer, kidney cancer, ovarian cancer, cervical cancer, sarcoma (SARC), liver cancer, and melanoma (**Supplement Figures S12** and **S13**). We analyzed the expression of the TGR5 gene in different cancer tissue from the HPA data set and the TCGA project. The results of the two databases are consistent (**Figure 3**). Therefore, based on the preliminary analysis results of the gene expression analysis, we found that there are significant differences in the expression of the TGR5 gene and protein among different tumors.

### Survival score and analysis show TGR5 has a significant effect on the survival prognosis of different tumors

**Figure 4:**
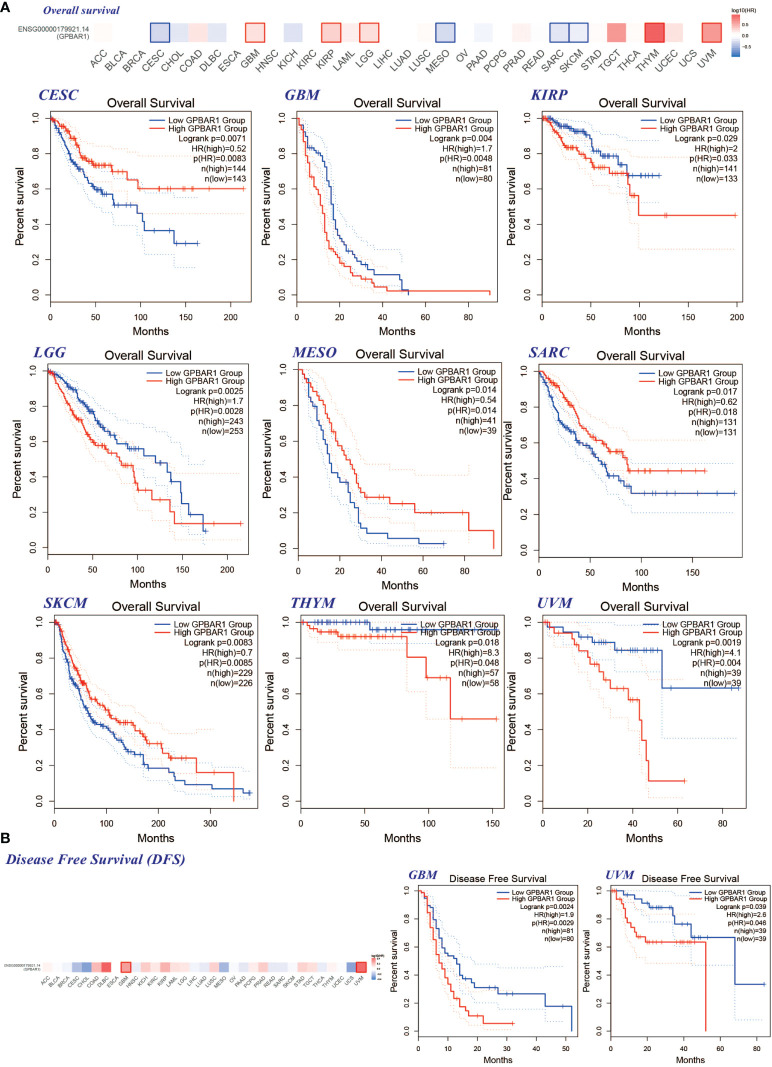
The correlation between the TGR5 gene expression level and patient survival in all TCGA tumors. **(A)** Overall survival in CESC, GBM, KIRP, LGG, MESO, SARC, SKCM, THYM, and UVM. **(B)** The GEPIA2 instrument is used to analyze the survival rate of GBM and UVM. This figure shows the survival plot and Kaplan–Meyer curve with positive results.

We divided tumors into high and low TGR5 expression groups and analyzed the correlation between TGR5 gene expression and survival scores obtained by TCGA and GEO data sets. As shown in **Figure 4**, it was apparent that the high expression of TGR5 was related to poor prognosis of OS for cancers such as GBM (p = 0.0048), KIRP (p = 0.033), lower grade glioma (LGG) (p = 0.0028), thymoma (THYM) (p = 0.048), and uveal melanoma (UVM) (p = 0.004). Then, the lower expression of TGR5 was linked with poor prognosis in CESC (p = 0.014), MESO (p = 0.048), SARC (p = 0.018), and SKCM (p = 0.0085) (**Figure 4A**). Data from the DFS analysis showed that high TGR5 gene expression was associated with poor prognosis of GBM (p = 0.0029) and UVM (p = 0.046) (**Figure 4B**).

Turning now to the experimental evidence on survival analysis using the Kaplan–Meier plotter tool, it can be seen that the lower expression of the TGR5 gene is correlated with poor relapse-free survival (RFS) (**Supplement Figure S5A**) (p = 0.0016), but there are no significant differences in poor OS, postprogression survival (PPS), and distant metastasis-free survival in breast cancer. For survival analysis of liver cancer, the lower expression of the TGR5 gene was linked with poor RFS (p = 0.021) and disease-specific survival (DSS) (p = 0.012). Besides, highly expressed TGR5 gene was related to poor OS (p < 0.0001), first progression (p < 0.0001), PPS (p < 0.0001) in gastric cancer, and correlated with poor OS (p = 0.016) in lung cancer, as well as poor PPS (p = 0.017) in ovarian cancer. The pan-cancer analysis also found that elevated TGR5 gene expression was related to poor RFS (p = 0.011) (**Supplement Figure S5A**). Together, these results provide important insights into the prognosis of TGR5 in several specific cancers.

### TGR5 was generally genetic hyperalteration and good prognosis in uterine corpus endometrial carcinoma and liver hepatocellular carcinoma patients

**Figure 5:**
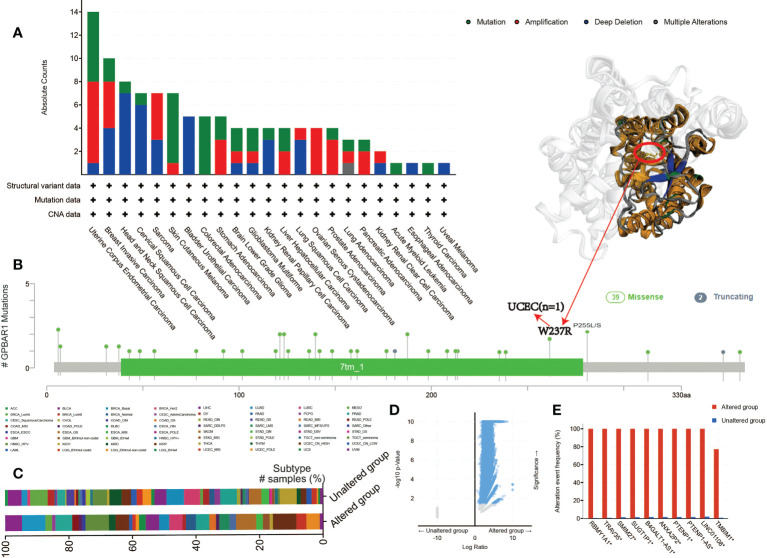
The mutation status of the GPBAR1 gene expression level in all TCGA tumors from the cBioPortal tool. The absolute count with mutation type **(A)** and mutation site **(B)** is presented. **(C–E)** Clinical outcomes **(C)** and genomic changes **(D, E)** in GPBAR1 gene alteration have also been analyzed.

Another aspect of cancer treatment that needs attention is genetic alterations. Therefore, we further investigate the TGR5 gene alteration in human tumor tissues. TGR5 has the largest change in uterine tumors, and its main type is mutation. Also, breast cancers have the highest in SARC with amplification as the primary type (**Figure 5A**). We also found that additional mutation and their location within TGR5 and no main type of genetic location alteration in **Figure 5B**. For example, W234R is a meaningless mutation found in only one case by UCEC. The position of W234R can be seen in the three-dimensional structure of the TGR5 protein. Besides, the clinical outcome and genomic change of TGR5 gene alteration have also been analyzed in **Figures 5C, D**. The alteration event frequency of the TGR5 gene expression in RBMY1A1, TRAV35, SMIM27, SUGT1P1, ANXA2P2, PTENP1, LINC01108, and TMBIM1 have been investigated in **Figure 5E**.

**Figure 6:**
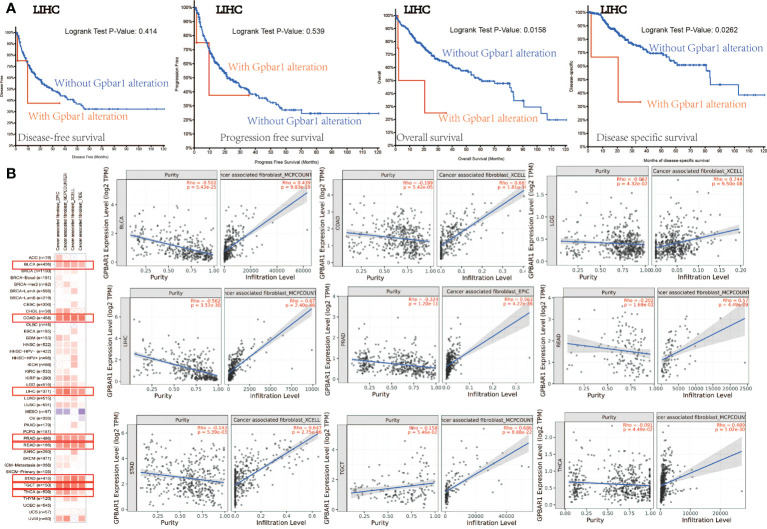
The mutation status with survival analysis and cancer-related fibroblast with TGR5 gene expression. **(A)** Use the cBioPortal tool to analyze the correlation between mutation status and OS (overall survival), DSS (disease-specific survival), DFS (disease-free survival), and PFS (progression-free survival). **(B)** A correlation analysis between TGR5 gene expression and cancer-related fibroblast, which include EPIC, MCPCOUNTER, XCELL, and TIDE across all TCGA tumors. Red means positive correlation (0–1), blue means negative correlation (−1 to 0). A value of p < 0.05 is considered statistically significant. Correlation values that are not statistically significant are indicated by crosses.

In addition, we analyzed the possible association between TGR5 gene alterations and the survival analysis of different cancers. In **Figure 6A**, we can see that LIHC cases with TGR5 gene alteration showed good prognosis in OS (p = 0.0158) and DSS (p = 0.0262) compared with cases without TGR5 gene alteration (**Figure 6A**). Besides, we also explored the correlation between TGR5 expression, microsatellite instability, and tumor mutational burden (TMB) across all tumors of TCGA; there is a negative correlation between TGR5 expression and tumor matrisome index (TMI) in ovarian serous cystadenocarcinoma (OV) (p = 0.019), pancreatic adenocarcinoma (PAAD) (p = 0.0042), STAD (p < 0.001), and COAD (p = 0.004) (**Supplement Figure S7D**). In additional, there is also a negative correlation between TGR5 expression and TMB in KIRP (p = 0.00093), LIHC (p = 0.043), COAD (p = 0.033), and STAD (p = 0.00086) but a positive correlation between TGR5 expression and LGG (p = 0.038) and LUSC (p = 0.01) (**Supplement Figure S7D**). The themes identified in these responses indicated that the TGR5 genetic alteration plays an important role in the prognosis of cases with different tumors and should be studied further.

### TGR5 was significantly correlated with immune infiltration

Immune infiltration plays an important role in the tumor microenvironment, which requires distinct immunotherapeutic interventions for maximal therapeutic effect. TGR5 also regulates fibroblast, macrophage innate immune, and T-cell cytokine activation in liver ischemia, and reperfusion injury and myasthenia gravis were reported in immune infiltration (6, 23–27). Herein, we first analyzed the immune microenvironment, immune cell score, immune pathway, and immune checkpoint and then study the possible relationship between the degree of immune filtration process of different immune cells in the TCGA tumor and the expression of the TGR5 gene (**Supplement Figures S7A, B, F, G**). The neoantigen result found that STAD (p = 0.0017) has negatively correlated with TGR5-related immune neoantigen (**Supplement Figure S7C**). The immune-related clinical outcome of the Cox model showed that GBM, KIRP, and LGG has significantly correlated with TGR5-related immune infiltration (**Supplement Figure S7H**). Second, we explore the EPIC, MCPCOUNTER, XCELL, TIDE, CIBEREORT, and CIBEREORT-ABS algorithms to investigate T-cell CD4+, cancer-associated fibroblasts, T-cell CD8+, T-cell regulators, and macrophages (**Figure 6B** and **Supplement Figures S8A**, **S9A**, **S10A** and **S11A**). The scatter graph data generated by the above tumor algorithm are shown in **Figure 6B** and **Supplement Figures S8B**, **S9B**, **S10B**, and **S11B**. For instance, the TGR5 expression level in UVM is negatively correlated with the infiltration level of the macrophage M2_TIDE (Cor = −0.541, p = 3.80e−07). The results of the correlational analysis were shown in the TGR5 gene expression and immune infiltration analysis.

### TGR5-related gene was correlated with osteoclast differentiation and Rap1 signaling pathway

**Figure 7:**
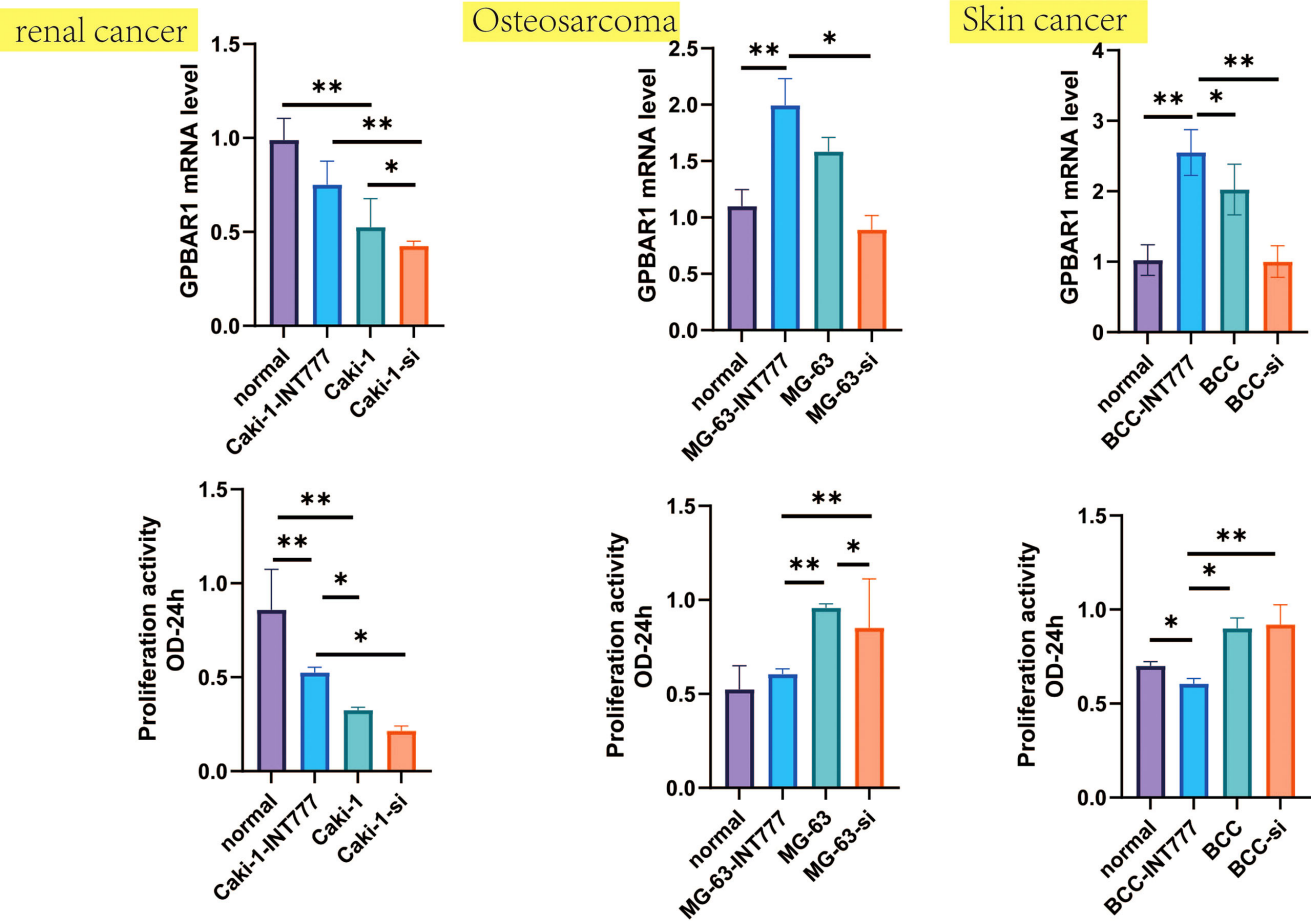
Proliferation activity and GPBAR1 mRNA were investigated by the CCK-8 test and the qPCR test, which compared normal renal cell, normal human osteoblasts, and normal skin cell with skin cancer, osteosarcoma, and renal cell carcinoma (each group: n = 8). *p < 0.05, **p < 0.01.

KEGG and GO enrichment analysis was shown between the two data sets. As shown in **Supplement Figure S19D** of KEGG data, it is found that “osteoclast differentiation”, “Rap1 signaling pathway”, and “regulation of actin cytoskeleton” may play an important role in TGR5-related tumorigenesis. The GO enrichment analysis data found that TGR5 may link with metabolism pathways, such as “regulation of hormone secretion”, “GPCR downstream signaling”, “drug induction of bile acid pathway”, “metabolism of steroids”, and others (**Figure 7**).

### DNA methylation analysis data

In the TCGA data set, TGR5 DNA methylation and the pathogenesis of various tumors were studied through MEXPRESS and SMART. First, we analyzed TGR5 DNA methylation in the distribution of different types of tumors such as BRCA, COAD, KIRC, LIHC, and LUAD (**Supplement Figure S14**) and then explore the chromosomal distribution of TGR5 DNA methylation probes and detailed genomic information of TGR5 in **Supplement Figures S15A, C**. Second, the CpG-aggregated methylation has found that all TCGA tumors have a higher level in BRCA, COAD, esophageal carcinoma (ESCA), KIRC, KIRP, LUAD, LUSC, prostate adenocarcinoma (PRAD), THCA (all ps < 0.001), BLCA, CESC, rectum adenocarcinoma (READ) (all ps < 0.01), and head and neck squamous cell carcinoma (HNSC) (p < 0.05) but a lower level in CHOL, LIHC, PAAD, pheochromocytoma and paraganglioma (PCPG) than in normal tissue (**Supplement Figure S15B**). Besides, we further analyzed the different probes with TGR5 DNA methylation in all TCGA tumors, which include eight probes such as cg20655350, cg18581950, and cg05728596 (**Supplement Figure S6**, **S16**, and **S17**). Also, clinical factors such as ethnicity, race, body mass index, age, and event were related to CpG island in UCEC patients and also were predicators for survival prognosis of TGR5 DNA methylation in LIHC, STAD, ESCA, and BLCA patients (**Supplement Figure S18**).

Besides, DNA methylation correlation, somatic mutation, copy number variations, and different tumor stages of TGR5 DNA methylation have also been analyzed by the SMART tool in **Supplement Figure S20**. For instance, LIHC has negatively correlated with probes such as cg20655350, cg18581950, cg06242216, cg25124402, cg02116437, cg22678065, and cg05728596 in gene methylation correlation and have 0.56% mutation in somatic mutation (**Supplement Figure S20A**).

### Cell viability and gene expression

We further used the CCK-8 and qPCR tests to analyze changes in cell activity and TGR5 gene expression between skin cancer, osteosarcoma and renal cell carcinoma, and normal cell lines. The results were consistent with the TCGA database, such as TGR5 was lowly expressed in renal cell carcinoma and highly expressed in skin cancer and osteosarcoma. At the same time, cell activity experiments have also found that TGR5 can increase the activity of renal cell carcinoma and reduce the activity of skin cancer and osteosarcoma cells (**Figure 7**).

## Discussion

Previous studies evaluated multifunctional TGR5—whether it can play an important role in the fundamental, cross-species cell biology process (28). TGR5 is not only a bile acid receptor but also the receptor for multiple receptors that plays an important role in many signaling pathways, for example, AKT (29). In our studies, we use the “HomoloGene” pathway analysis, DNA methylation analysis, and immune infiltration data to show that TGR5 plays a potential role across different species. These results also further support the idea that TGR5 may be a possible target for tumor therapy in different species.

The most important clinically and experimentally relevant finding has been investigated: the relationship between TGR5 and clinical outcome, particularly tumors (9–12, 30–37). Whether TGR5 has a certain molecular mechanism in all TCGA tumors needs to be further clarified. Through a literature search, we found that there is currently a lack of pan-cancer analysis to explore the role of TGR5 in different tumors. Therefore, based on TCGA, cBioPortal, GEPIA2, and HEPIA, we analyzed TGR5 gene expression, protein levels, gene changes, DNA methylation, and survival analysis of 33 TCGA tumors.

The TGR5 gene has obvious expression differences in different tumor tissues. We found that TGR5 has high expression in tumors of BLCA, BRCA, lymphoid neoplasm diffuse large B-cell lymphoma (DLBC), KICH, LUAD, LUSC, and THYM but lower expression in tumors of acute myeloid leukemia (LAML), PAAD, and CHOL than normal tissue. Besides, we further found that a low expression of TGR5 also has better OS in GBM, KIRP, LGG, THYM, and UVM but a high expression related with better OS in CESC, MESO, SARC, and SKCM, which means that TGR5 may be an important indicator for predicting the prognosis of cancer patients. Besides, TGR5 also plays different roles in different tumors, which is also consistent with our research results (37–39). Our cellular experiments and TGR5 gene expression levels in skin cancer, osteosarcoma, and renal cell carcinoma have further validated the results. In addition, we found that the increase in TGR5 expression in THYM is consistent with the poor prognosis of OS. The role of TGR5 in thymic cancer has not yet been reported, so our study may provide an important biological marker for the diagnosis of THYM.

The role of TGR5 in different tumor tissues is obviously different. For example, TGR5 can increase the activity of renal cell carcinoma but reduce the activity of skin cancer and osteosarcoma. In many studies, TGR5 is one of the most common therapeutic targets against hepatocellular carcinoma by regulating energy homeostasis and glucose metabolism. *In vivo*, TGR5 deficiency in mice promoted diethylpromazine-induced hepatocyte death, compensatory proliferation, gene expression of certain inflammatory cytokines, matrix metalloproteinases, and liver carcinogenesis than wild-type mice. *In vitro*, TGR5 activation strongly inhibits hepatocellular carcinoma proliferation and migration by inhibiting STAT3 signaling and its DNA-binding activity^6,7^. Therefore, TGR5 receptor could be a new potential biomarker for the diagnosis and treatment of hepatocellular carcinoma in the future. Besides, TGR5 activation can inhibit the proliferation and migration of gastric cancer cells by inhibiting STAT3 and NF-κB signaling pathways^8^. In HCT116 cells, SW480 cells, and DSS-induced CRC mice, ursodeoxycholic acid (UDCA), as one of the main active components of bile, inhibits the malignant progression of colorectal cancer through TGR5-mediated cAMP-PKA-RhoA signaling pathway antagonizing YES-associated protein^9^. However, in lung cancer, binding and activation of TGR5 in H1299 lung cancer cells can increase the content of cAMP and the phosphorylation levels of protein kinase A (PKA)^10^. TGR5 activation strongly inhibited JAK2-STAT3 signaling *in vitro* and *in vivo*. The activation of TGR5 in the mesangial membrane of non–small cell lung cancer cells mediates JAK2-STAT3 signaling pathway, which exacerbates the development and migration of lung tumor cells^6^. Tgr5-induced cAMP-PKA-CREB and JAK2-STAT3 signaling pathways are promising therapeutic strategies and predict the efficacy of lung cancer treatment

For breast cancer, we investigate the data set of TCGA-BRCA and found a correlation between high TGR5 gene expression in tumors and clinical stages, a good RFS prognosis for BRCA. Overexpression of TGR5 has antiproliferation and pro-apoptosis effects on breast cancer cell adipogenesis (9). However, this outcome is contrary to that of Min-Chan Chen et al. (2016), who found that the high expression of the TGR5 gene is an indicator of poor prognosis for patients with gastric cancer and breast cancer (40). We used the same database to analyze and found that the survival results were different compared with Chen et al.’s studies, and a possible explanation for this might be that the sample size was increased.

According to our studies, the high TGR5 gene expression in lung and gastric cancers raises the possibility of poor OS and positive correlation of TMB. TGR5 promotes the growth and migration of non–small cell lung cancer cells through the JAK2-STAT3 signaling pathway (12). Cao et al. found that a moderate or high TGR5 expression is associated with the decreased survival rate of patients with gastric adenocarcinoma (30), which may link with antagonizing STAT3 and NF-κB signaling pathway (41, 42). However, for liver cancer, the high expression of the TGR5 gene also associated with good RFS and DSS and negative correlation of TMB, which matches those observed in earlier studies (13). Moreover, high TGR5 gene expression also correlated with good OS in skin cancer. This was also similar to our earlier observations, which showed that high expression of TGR5 plays a positive role in skin disease, which can reverse the development of alopecia aureate (8).

Abnormal DNA methylation is considered to be a key event in the occurrence and development of cancer and related to chromatin remodeling and abnormal gene expression in many malignant tumors (43). In our study, the high TGR5 DNA methylation in the liver and esophageal cancer was correlated with different clinical stages. This finding broadly supports the work of other studies in this area, linking TGR5 DNA methylation with liver cancer (e.g., Han et al. and Gao et al.) (32, 44). In addition, there are similarities between the attitudes expressed by Chen et al. in this study, and those described that Cox regression analysis generates risk models for GPBAR1 and esophageal adenocarcinoma (45). Besides, we have only selected to analyze the methylation levels and prognosis of four cancers, namely, LIHC, STAD, ESCA and BLCA, and we will further supplement other tumor categories in future studies.

The role and underlying mechanism of TGR5 in regulating immune infiltration play an important role in multiple diseases (27). We also found the immune pathways and immune microenvironment of TGR5 in all TCGA tumor cells and found that TGR5 was also significantly correlated with immune infiltration. These results corroborate the findings of a great deal of the previous work in cancer-related fibroblast, macrophage innate immune, and T-cell cytokine activation (6, 46, 47). In addition, the KEGG/GO pathway analysis found “regulation of hormone secretion”, “Rap1 pathway”, “osteoclast differentiation”, and “drug induction of bile acid pathway” among the top hits. Comparison of the findings with those of other studies confirms that TGR5 may play an important role in hormone secretion, which can affect the progress of multimetabolic diseases (48–50). Besides, TGR5 receptor activation may be associated with the relaxation of gastric smooth muscle by the Rap1 pathway (51). These results are in accordance with recent studies indicating that TGR5 plays an important role in bone metabolism and bile acid metabolism (52, 53).

## Conclusion

Overall, this study from pan-cancer analysis strengthens the idea of a statistical correlation of TGR5 with gene expression, clinical prognosis, genetic alteration, tumor mutation burden, immune cell infiltration, and microsatellite instability for all TCGA tumors. Therefore, this study provides broad molecular signatures for further functional and therapeutic studies of TGR5 and also represents a systemic approach to characterize key proteins in cancer.

## Data availability statement

The original contributions presented in the study are included in the article/**Supplementary Material**. Further inquiries can be directed to the corresponding authors.

## Author contributions

Conception and design: ZYG and TK. Acquisition, analysis, and interpretation of the data: ZYG, SFL, ZW, QGZ, LYL, and ZQG. Drafting and writing: XJ and KT. Final approval of the article: ZYG, SFL, ZW, QGZ, LYL, KT and ZQG. All authors contributed to article and approved the submitted version.

## Funding

This work was supported by grants from the Clinical Research Project of Shanghai Tenth People’s Hospital (YNCR2C027) and Research Fund of Shanghai Tongren Hospital, Shanghai Jiaotong University School of Medicine (No: TRYJ2021JC02) and Tongren Xinxing (TRKYRC-xx202215).

## Acknowledgments

We would like to thank all participants in this study.

## Conflict of interest

The authors declare that the research was conducted in the absence of any commercial or financial relationships that could be construed as a potential conflict of interest.

## Publisher’s note

All claims expressed in this article are solely those of the authors and do not necessarily represent those of their affiliated organizations, or those of the publisher, the editors and the reviewers. Any product that may be evaluated in this article, or claim that may be made by its manufacturer, is not guaranteed or endorsed by the publisher.

## Abbreviations

GPBAR1, G protein–coupled bile acid receptor 1; GEO, Gene Expression Integration; HPA, Human Protein Atlas; TCGA, Tumor Genome Atlas; OS, overall survival; ERK, extracellular signal-regulated kinases; DFS, disease-free survival; CAN, copy number change; DMEM, Dulbecco’s modified eagle medium; BBC, basal cell carcinoma; ACC, adrenocortical carcinoma; BLCA, bladder urothelial carcinoma; BRCA, breast invasive carcinoma; CESC, cervical squamous cell carcinoma and endocervical adenocarcinoma; CHOL, cholangiocarcinoma (bile duct); COAD, colon adenocarcinoma; DLBC, lymphoid neoplasm diffuse large B-cell lymphoma; ESCA, esophageal carcinoma; GBM, glioblastoma multiforme; HNSC, head and neck squamous cell carcinoma; KICH, kidney chromophobe; KIRC, kidney renal clear cell carcinoma; KIRP, kidney renal papillary cell carcinoma; LAML, acute myeloid leukemia; LGG, brain lower grade glioma; LIHC, liver hepatocellular carcinoma; LUAD, lung adenocarcinoma; LUSC, lung squamous cell carcinoma; OV, ovarian serous cystadenocarcinoma; PAAD, pancreatic adenocarcinoma; PCPG, pheochromocytoma and paraganglioma (adrenal gland); PRAD, prostate adenocarcinoma; READ, rectum adenocarcinoma; SARC, sarcoma; SKCM, skin cutaneous melanoma; STAD, stomach adenocarcinoma; TGCT, testicular germ cell tumors; THCA, thyroid carcinoma; THYM, thymoma; UCEC, uterine corpus endometrial carcinoma; UCS, uterine carcinosarcoma; UVM, uveal melanoma.

## Glossary

**Table:**

GPBAR1	G protein&ndash;coupled bile acid receptor 1
GEO	Gene Expression Integration
HPA	Human Protein Atlas
TCGA	Tumor Genome Atlas
OS	overall survival
ERK	extracellular signal-regulated kinases
DFS	disease-free survival
CAN	copy number change
DMEM	Dulbecco&rsquo;s modified eagle medium
BBC	basal cell carcinoma
ACC	adrenocortical carcinoma
BLCA	bladder urothelial carcinoma
BRCA	breast invasive carcinoma
CESC	cervical squamous cell carcinoma and endocervical adenocarcinoma
CHOL	cholangiocarcinoma (bile duct)
COAD	colon adenocarcinoma
DLBC	lymphoid neoplasm diffuse large B-cell lymphoma
ESCA	esophageal carcinoma
GBM	glioblastoma multiforme
HNSC	head and neck squamous cell carcinoma
KICH	kidney chromophobe
KIRC	kidney renal clear cell carcinoma
KIRP	kidney renal papillary cell carcinoma
LAML	acute myeloid leukemia
LGG	brain lower grade glioma
LIHC	liver hepatocellular carcinoma
LUAD	lung adenocarcinoma
LUSC	lung squamous cell carcinoma
OV	ovarian serous cystadenocarcinoma
PAAD	pancreatic adenocarcinoma
PCPG	pheochromocytoma and paraganglioma (adrenal gland)
PRAD	prostate adenocarcinoma
READ	rectum adenocarcinoma
SARC	sarcoma
SKCM	skin cutaneous melanoma
STAD	stomach adenocarcinoma
TGCT	testicular germ cell tumors
THCA	thyroid carcinoma
THYM	thymoma
UCEC	uterine corpus endometrial carcinoma
UCS	uterine carcinosarcoma
UVM	uveal melanoma
