# Supplementary figures and images for: The role of TGR5 as an onco-immunological biomarker in tumor staging and prognosis by encompassing the tumor microenvironment
Source: Front Oncol. 2022 Oct 20;12:953091. doi: 10.3389/fonc.2022.953091 (PMC9630950; doi:10.3389/fonc.2022.953091)

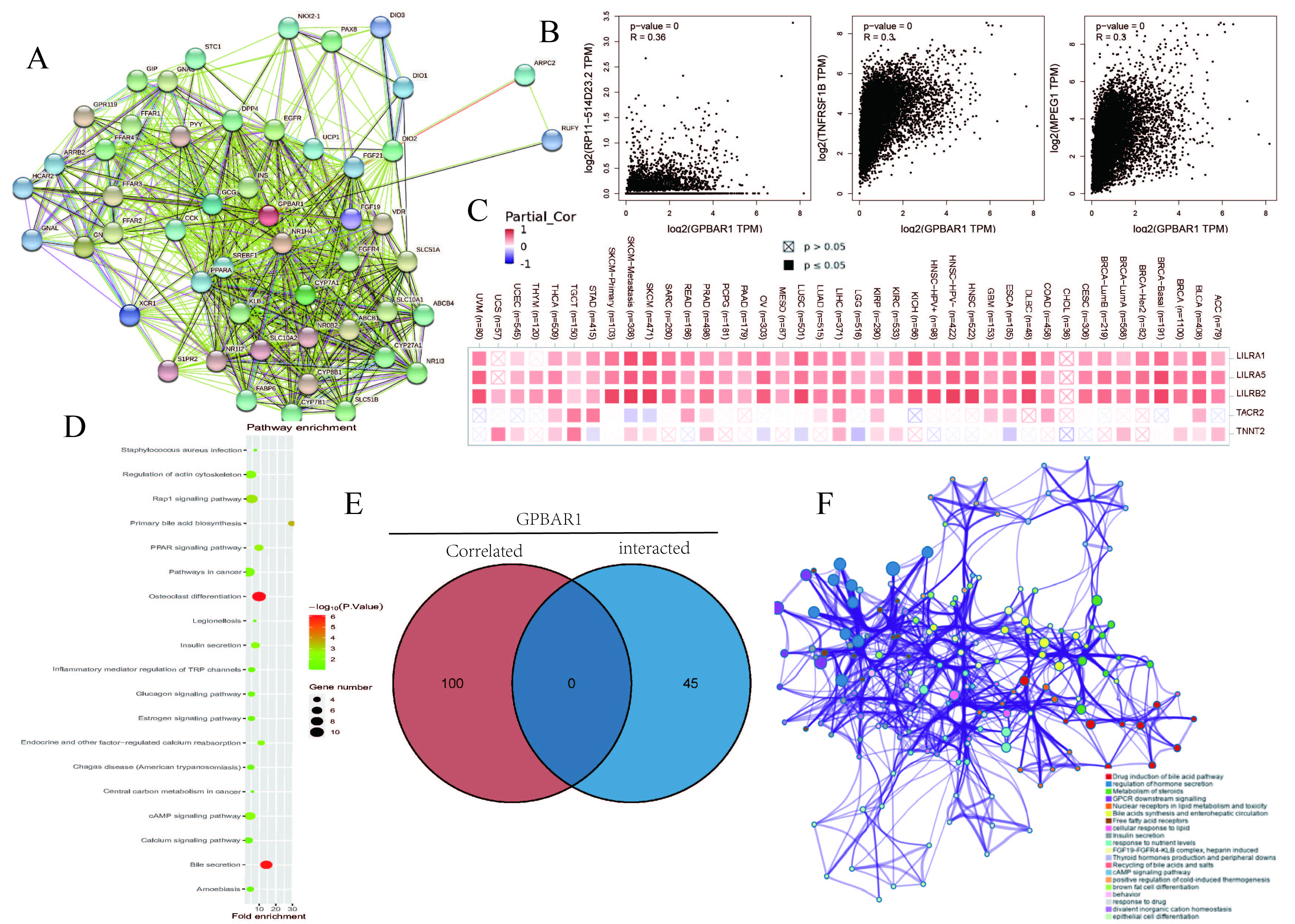

Supplement: Supplementary file 1 [file Image_1.jpeg]

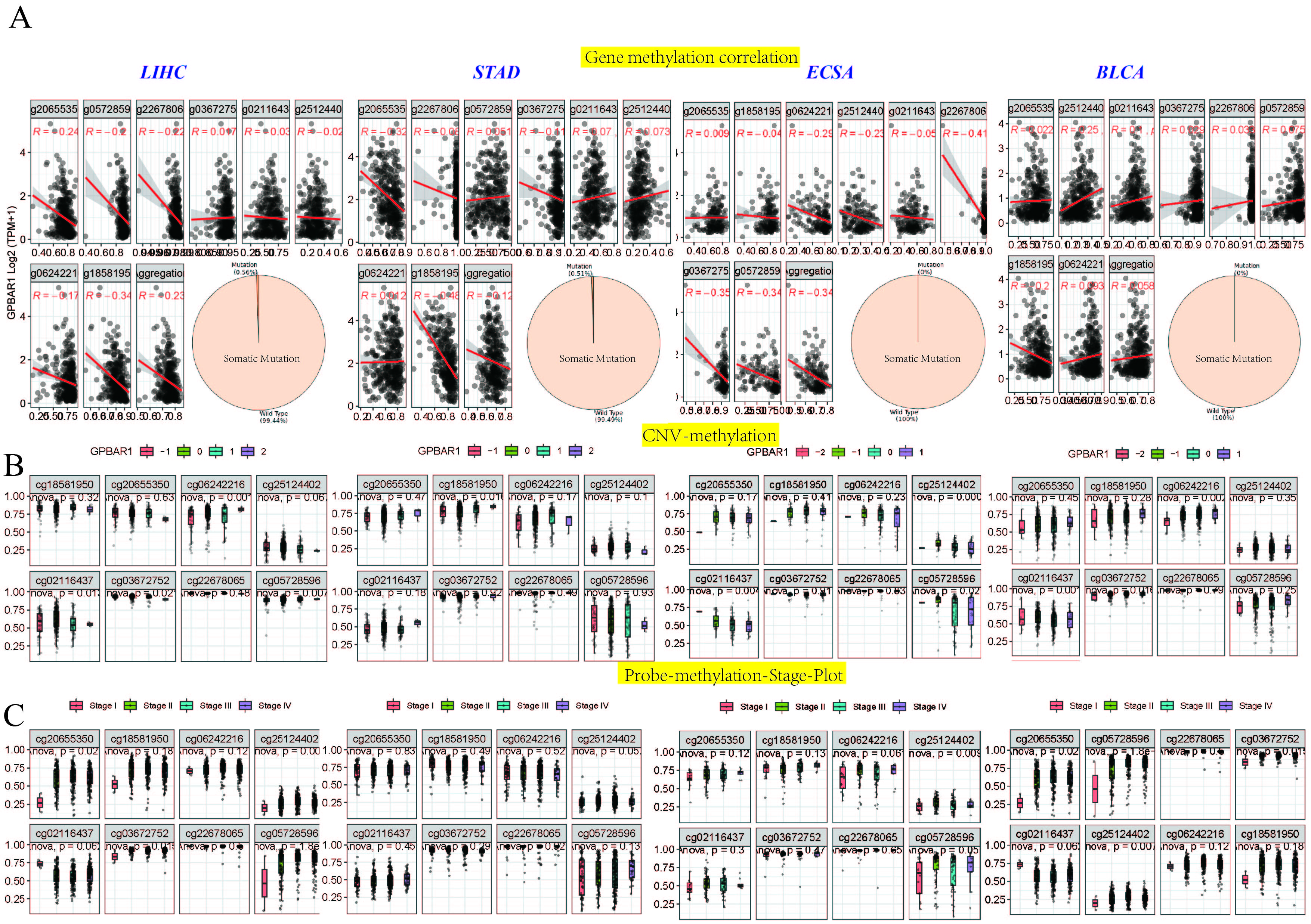

Supplement: Supplementary file 2 [file Image_2.jpeg]

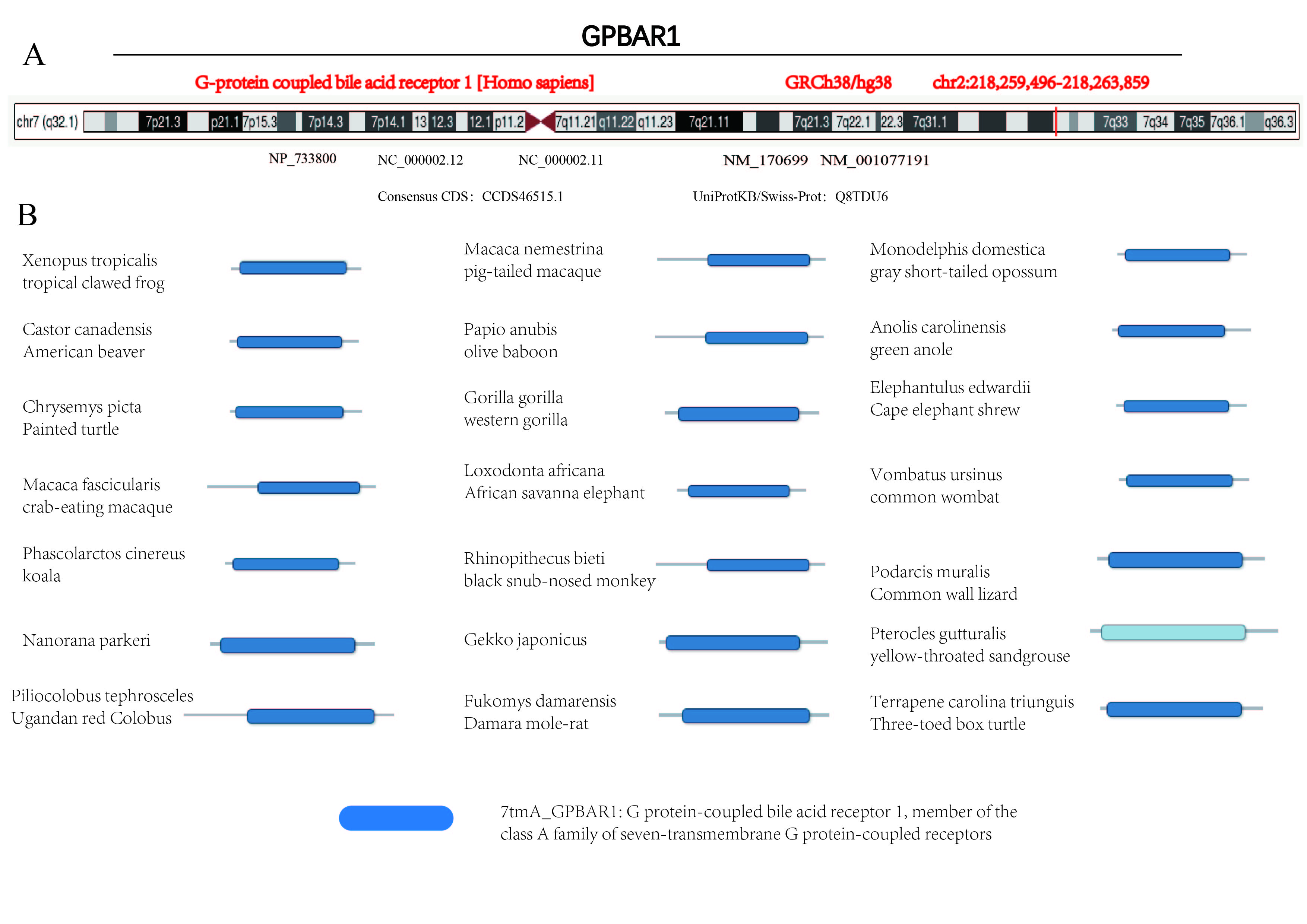

Supplement: Supplementary file 3 [file Image_3.jpeg]

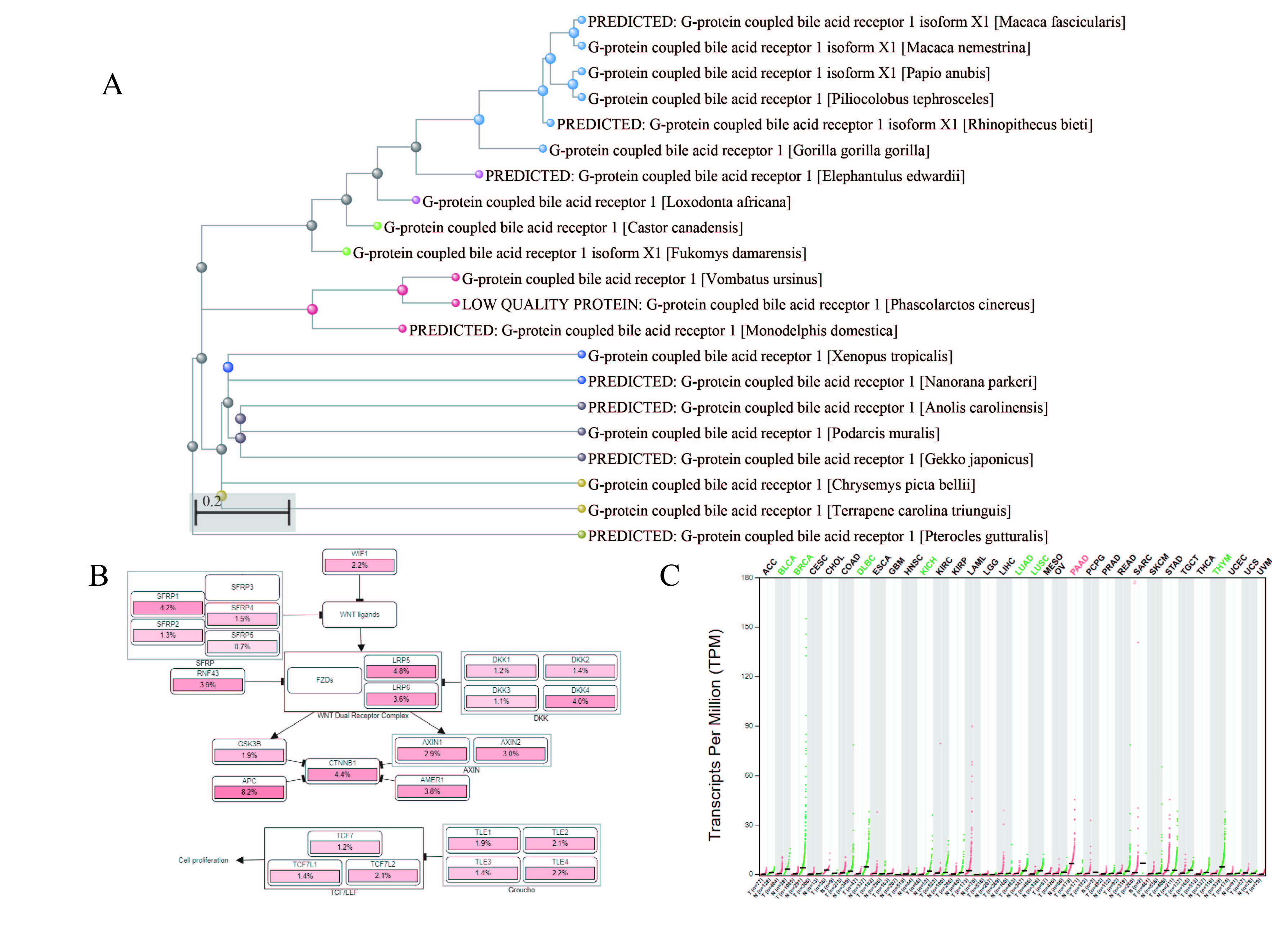

Supplement: Supplementary file 4 [file Image_4.jpeg]

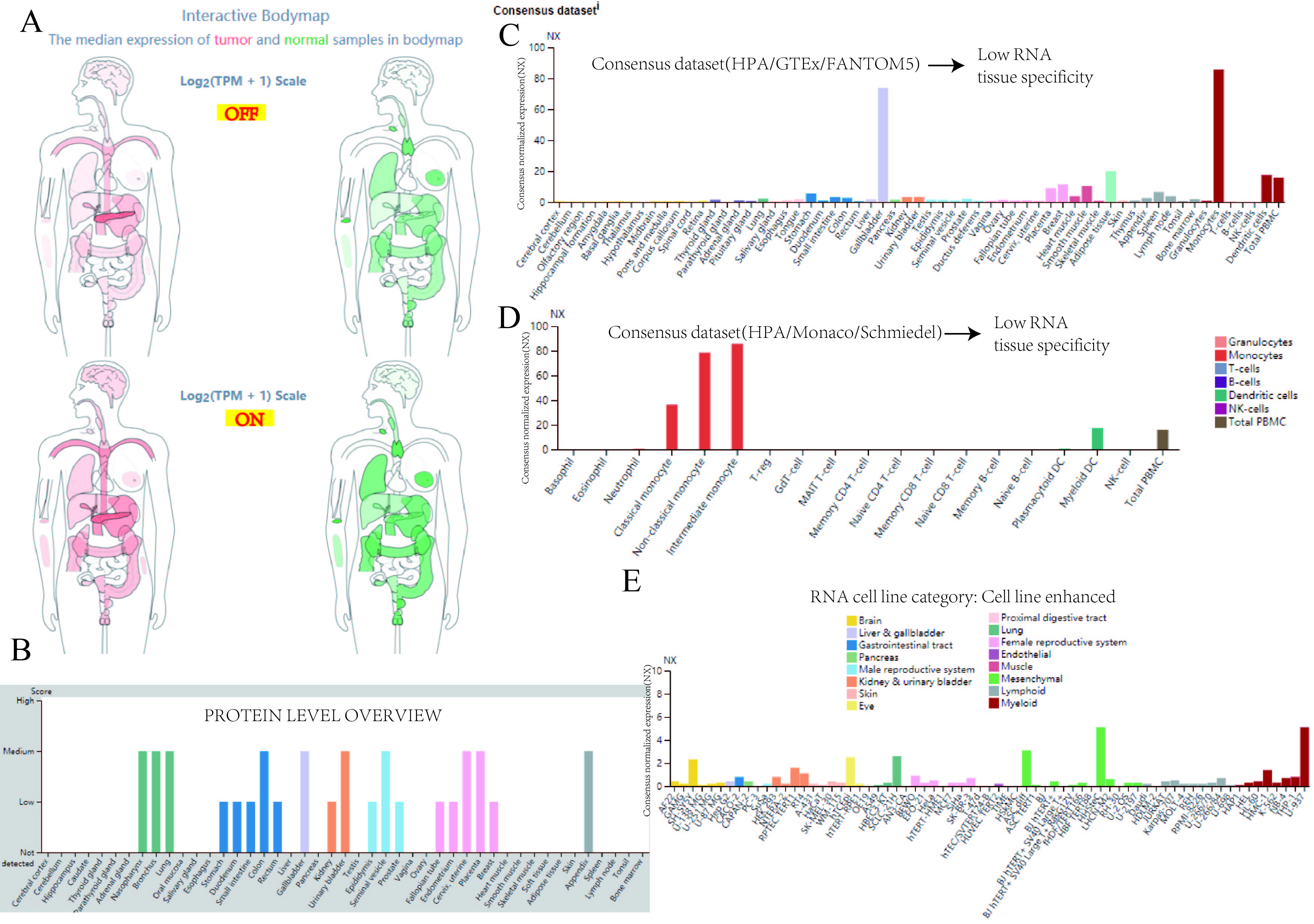

Supplement: Supplementary file 5 [file Image_5.jpeg]

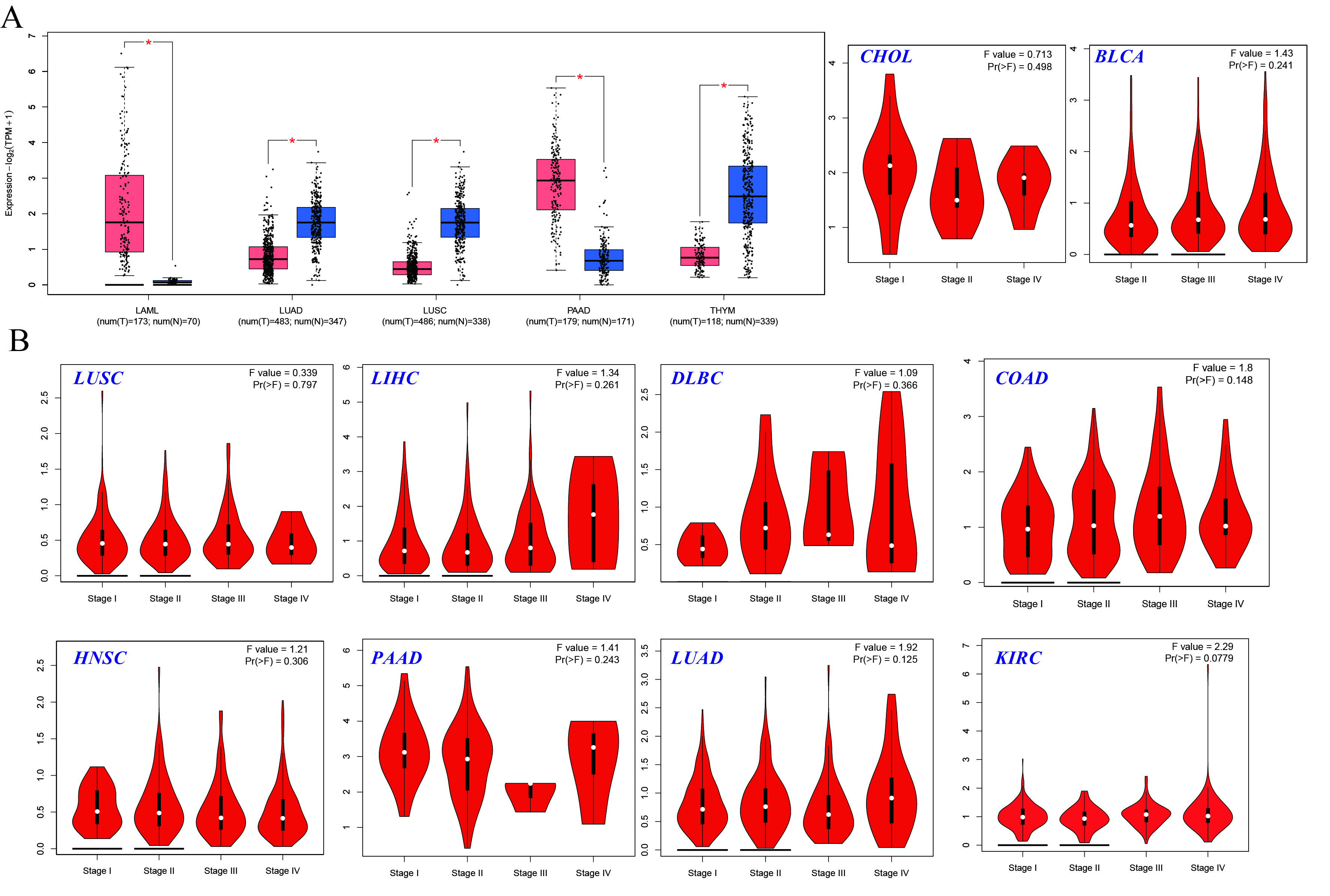

Supplement: Supplementary file 6 [file Image_6.jpeg]

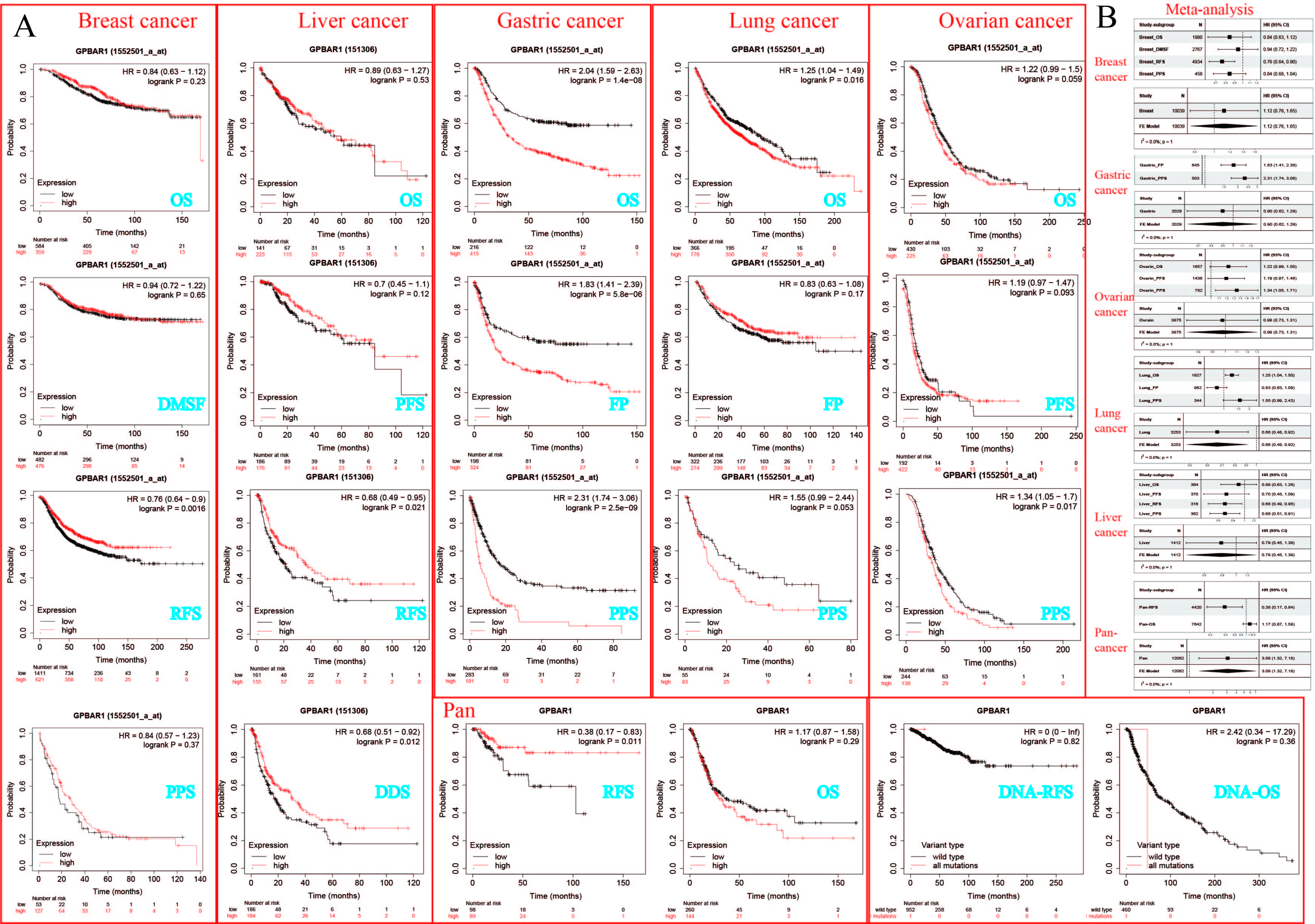

Supplement: Supplementary file 7 [file Image_7.jpeg]

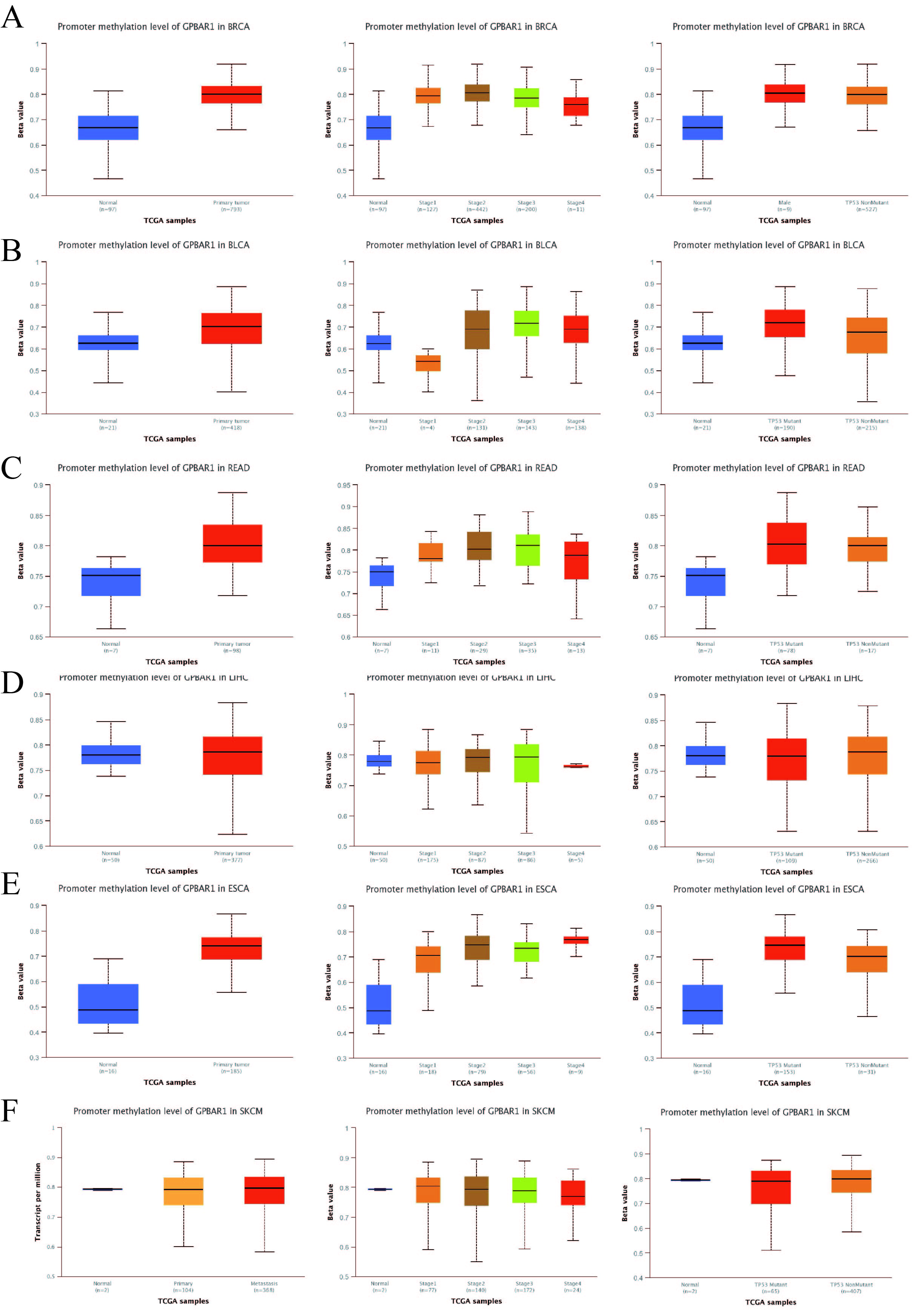

Supplement: Supplementary file 8 [file Image_8.jpeg]

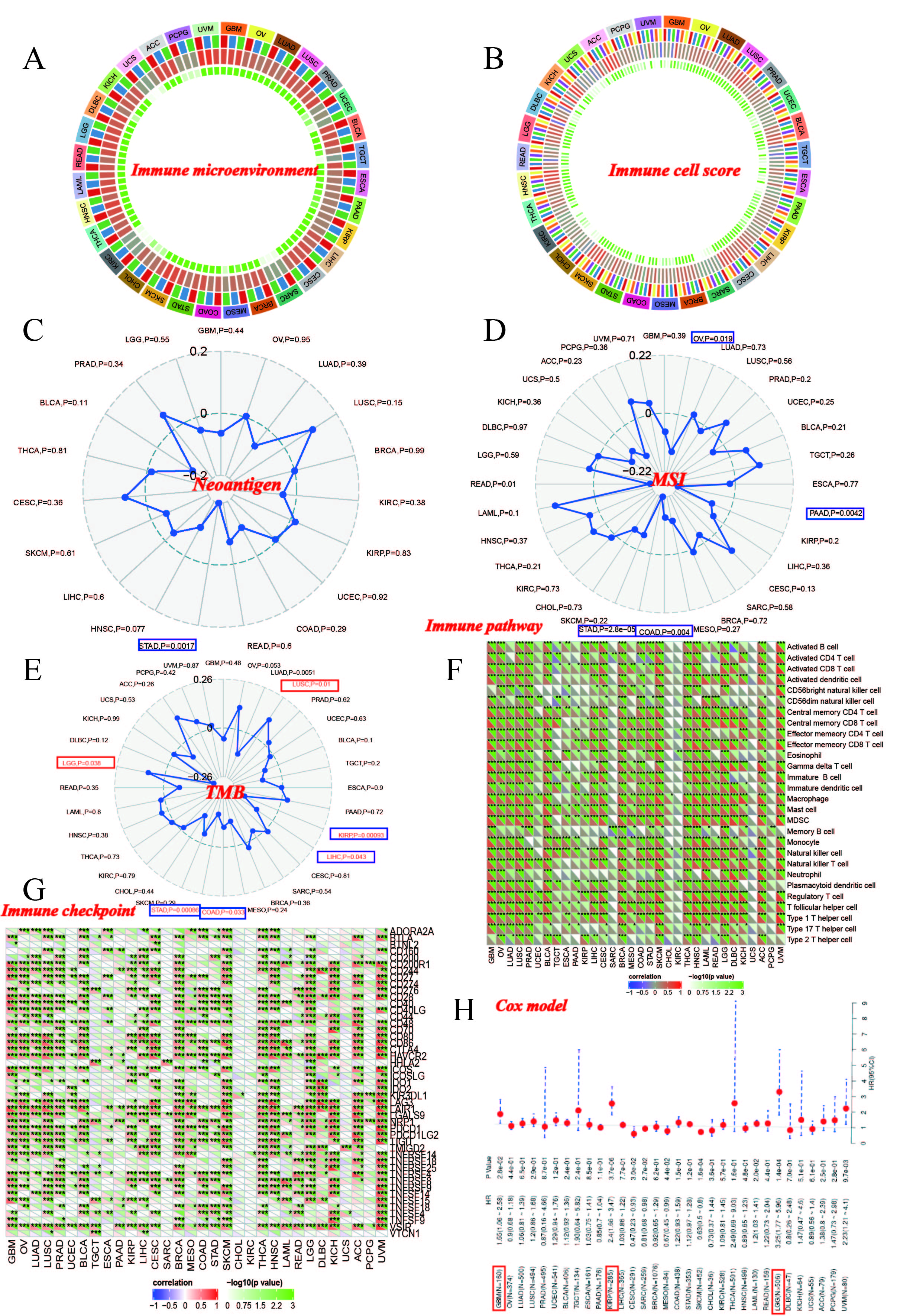

Supplement: Supplementary file 9 [file Image_9.jpeg]

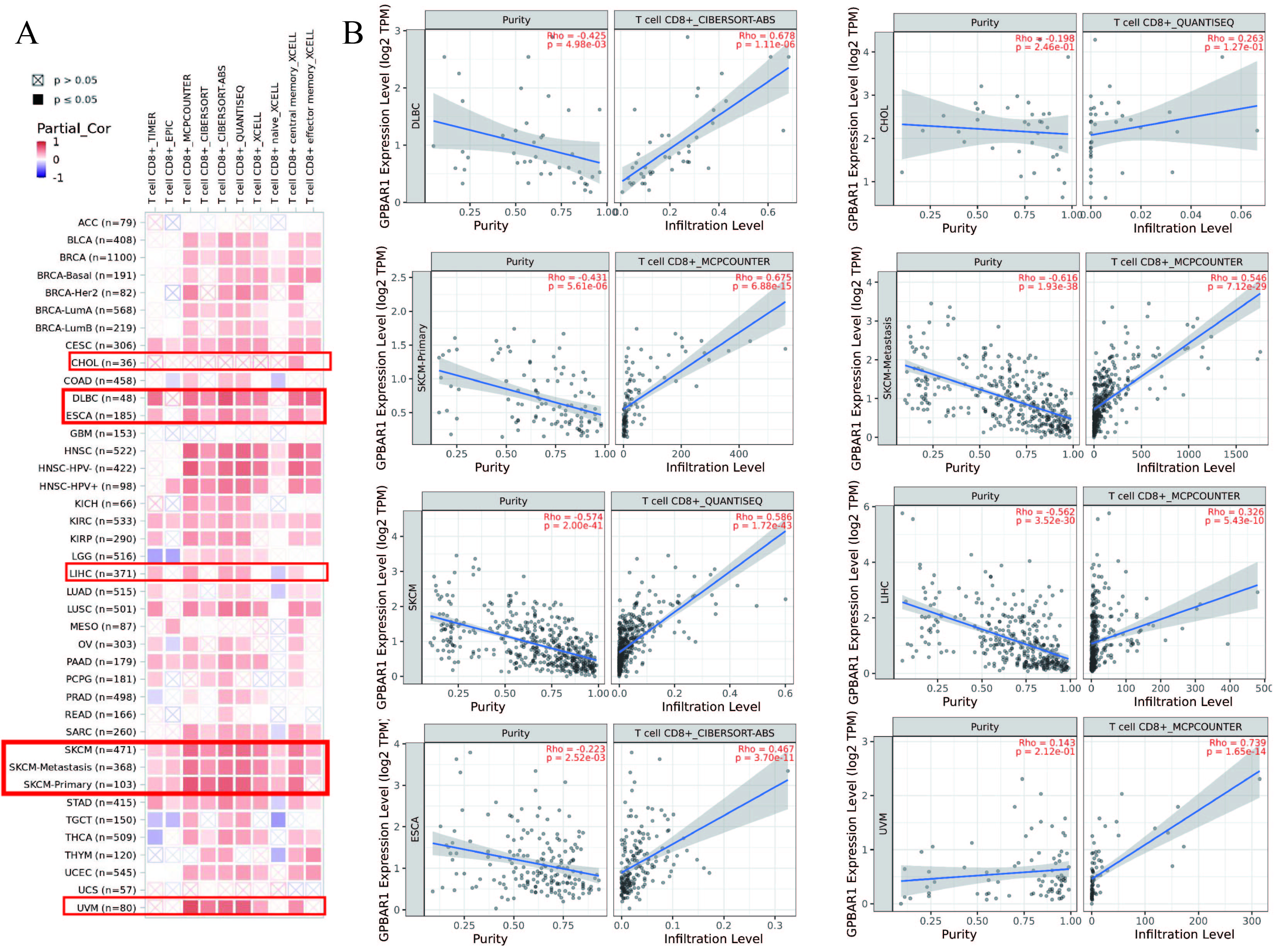

Supplement: Supplementary file 10 [file Image_10.jpeg]

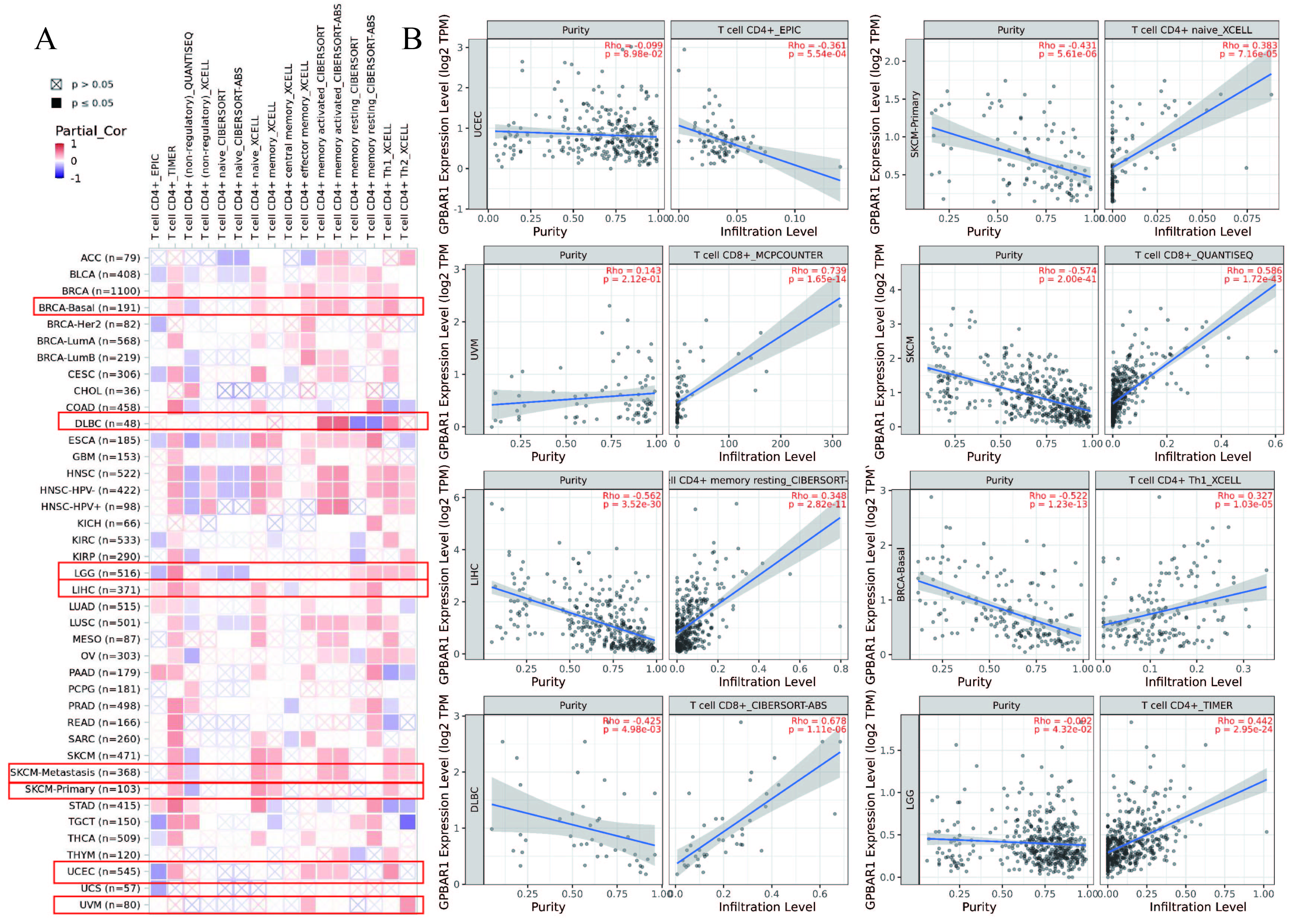

Supplement: Supplementary file 11 [file Image_11.jpeg]

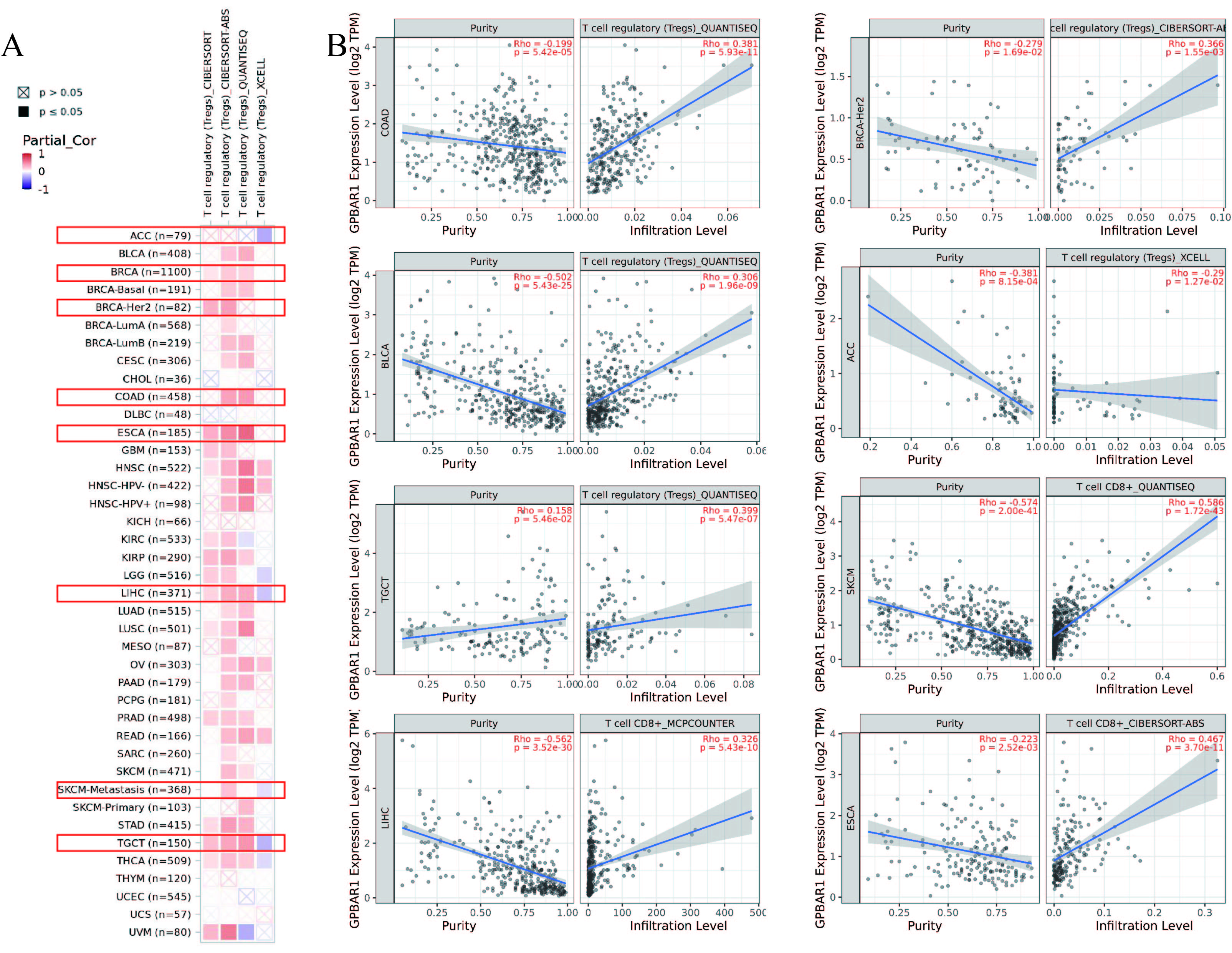

Supplement: Supplementary file 12 [file Image_12.jpeg]

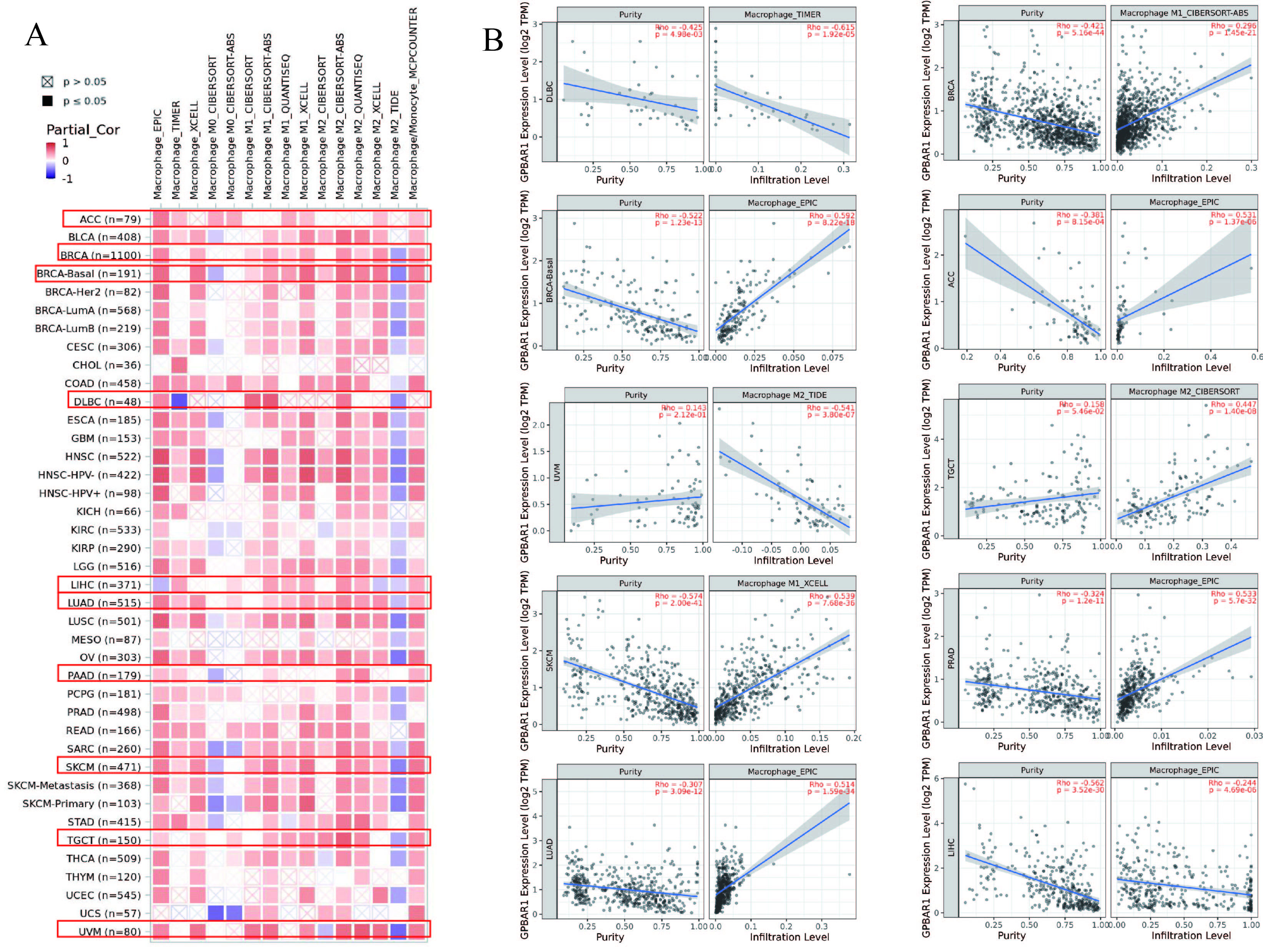

Supplement: Supplementary file 13 [file Image_13.jpeg]

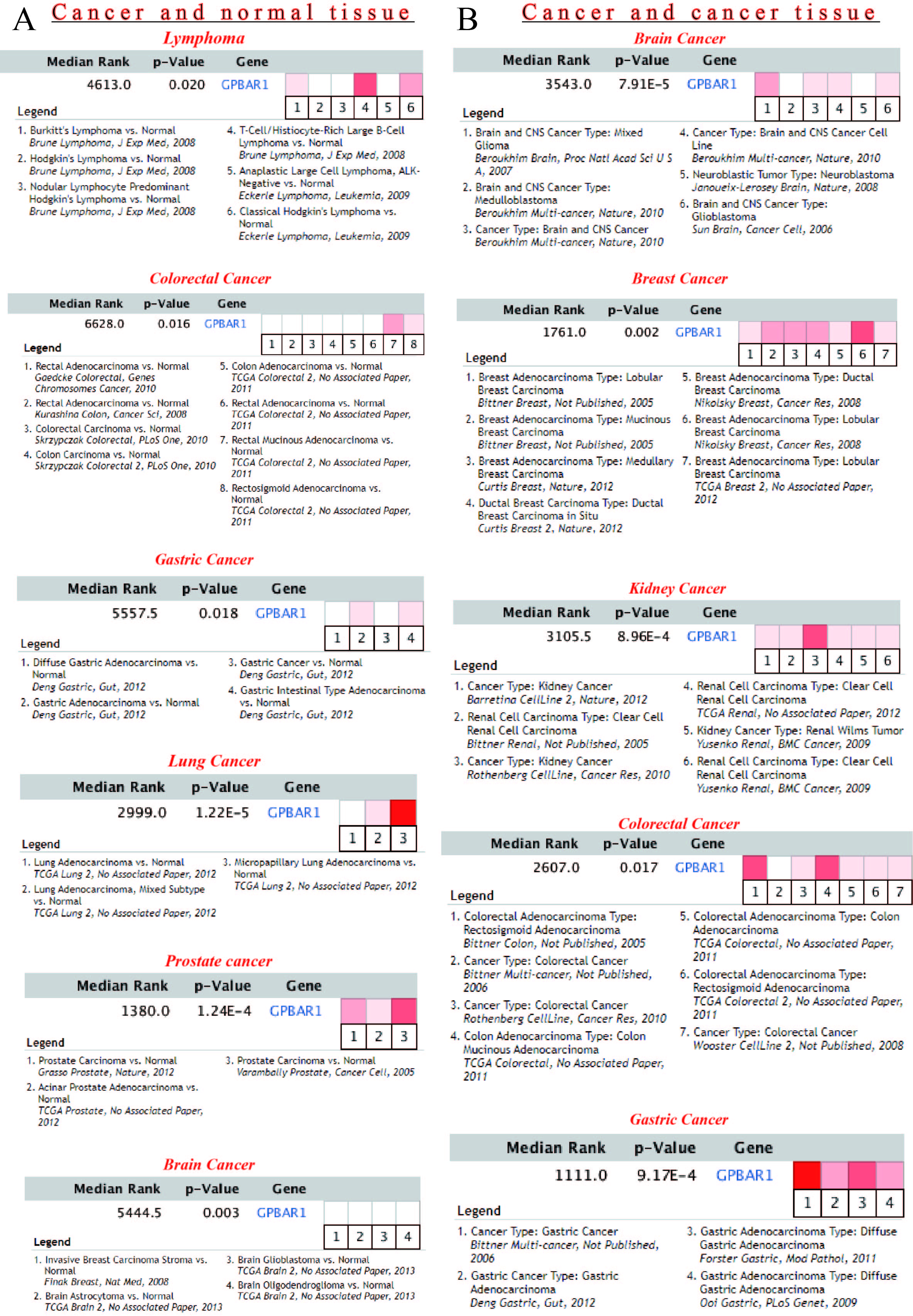

Supplement: Supplementary file 14 [file Image_14.jpeg]

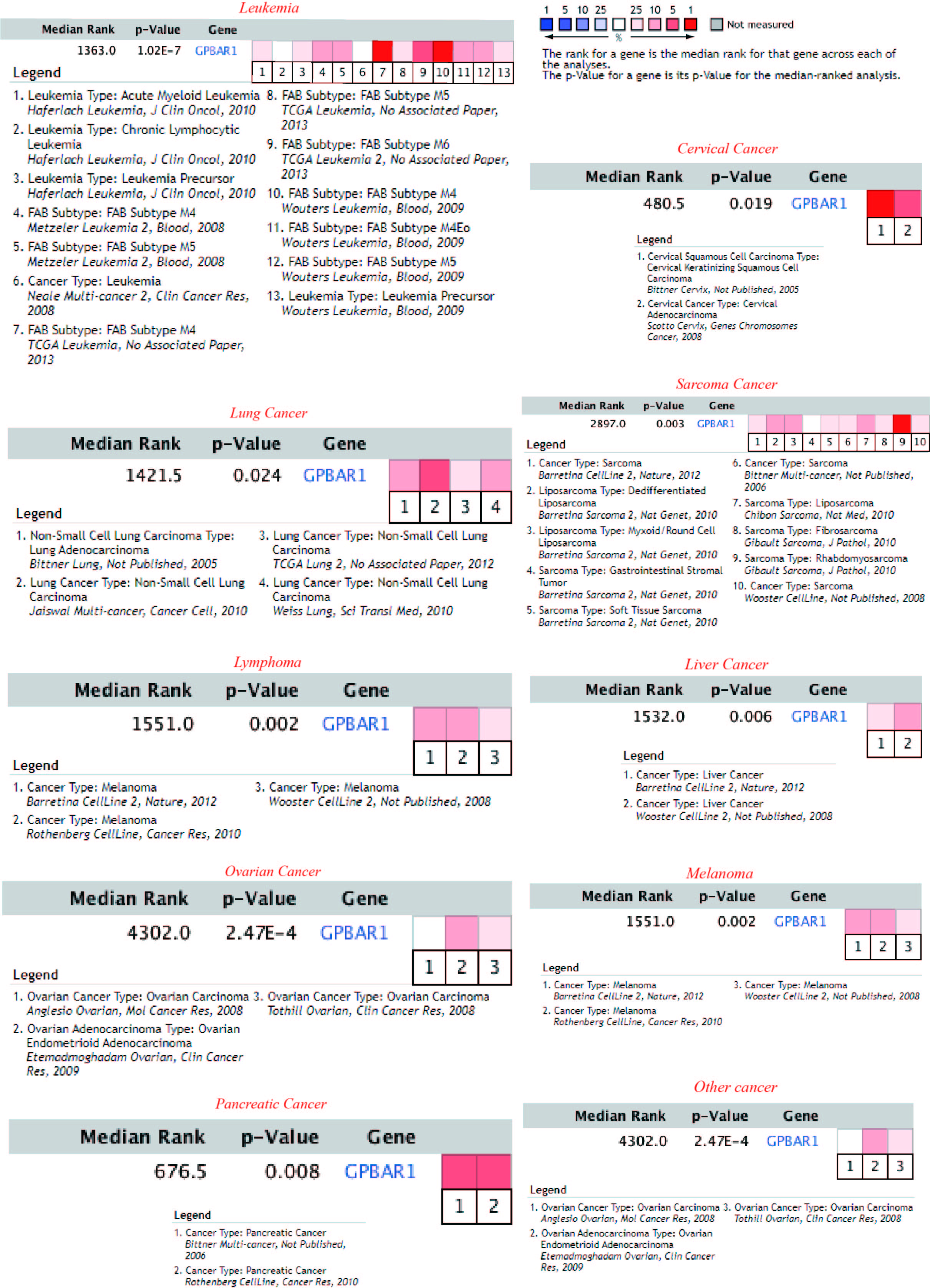

Supplement: Supplementary file 15 [file Image_15.jpeg]
